# The T Box Transcription Factor TBX2 Promotes Epithelial-Mesenchymal Transition and Invasion of Normal and Malignant Breast Epithelial Cells

**DOI:** 10.1371/journal.pone.0041355

**Published:** 2012-07-23

**Authors:** Bin Wang, Linsey E. Lindley, Virneliz Fernandez-Vega, Megan E. Rieger, Andrew H. Sims, Karoline J. Briegel

**Affiliations:** 1 Department of Biochemistry and Molecular Biology, Braman Family Breast Cancer Institute, Sylvester Comprehensive Cancer Center, University of Miami Miller School of Medicine, Miami, Florida, United States of America; 2 Applied Bioinformatics of Cancer Research Group, Breakthrough Research Unit, Edinburgh Cancer Research Centre, Edinburgh, United Kingdom; University Medical Center Utrecht, Netherlands

## Abstract

The T box transcription factor TBX2, a master regulator of organogenesis, is aberrantly amplified in aggressive human epithelial cancers. While it has been shown that overexpression of TBX2 can bypass senescence, a failsafe mechanism against cancer, its potential role in tumor invasion has remained obscure. Here we demonstrate that TBX2 is a strong cell-autonomous inducer of the epithelial-mesenchymal transition (EMT), a latent morphogenetic program that is key to tumor progression from noninvasive to invasive malignant states. Ectopic expression of TBX2 in normal HC11 and MCF10A mammary epithelial cells was sufficient to induce morphological, molecular, and behavioral changes characteristic of EMT. These changes included loss of epithelial adhesion and polarity gene (E-cadherin, ß-catenin, ZO1) expression, and abnormal gain of mesenchymal markers (N-cadherin, Vimentin), as well as increased cell motility and invasion. Conversely, abrogation of endogenous TBX2 overexpression in the malignant human breast carcinoma cell lines MDA-MB-435 and MDA-MB-157 led to a restitution of epithelial characteristics with reciprocal loss of mesenchymal markers. Importantly, TBX2 inhibition abolished tumor cell invasion and the capacity to form lung metastases in a Xenograft mouse model. Meta-analysis of gene expression in over one thousand primary human breast tumors further showed that high *TBX2* expression was significantly associated with reduced metastasis-free survival in patients, and with tumor subtypes enriched in EMT gene signatures, consistent with a role of TBX2 in oncogenic EMT. ChIP analysis and cell-based reporter assays further revealed that TBX2 directly represses transcription of *E-cadherin*, a tumor suppressor gene, whose loss is crucial for malignant tumor progression. Collectively, our results uncover an unanticipated link between TBX2 deregulation in cancer and the acquisition of EMT and invasive features of epithelial tumor cells.

## Introduction

The developmentally important transcription factor T box 2 (TBX2) is abnormally amplified with high prevalence in aggressive human epidermal cancers. TBX2 maps to chromosome 17q23.2, a region that is aberrantly amplified in over 40% of breast cancers [1], 40% of melanomas [2], 40% of ovarian [3], 56% of endometrial [4], and 60% of pancreatic cancers [5], correlating with poor clinical outcome. In breast cancer, *TBX2* gene amplification is associated with invasive hereditary BRCA1- and BRCA2-related cancers, high-grade-sporadic breast tumors, and distant metastases [6], [7], [8]. TBX2 is also amplified and overexpressed in several human breast carcinoma cell lines [6], [9], [10]. However, the potential role of TBX2 in malignant tumor progression has remained unclear.

TBX2 is a member of the evolutionary conserved T box transcription factor family [11], [12], a class of master regulators of embryogenesis that share a T box DNA binding domain and comprise many disease genes [13]. TBX2 acts mainly as a transcriptional repressor [14], [15], [16] that has been shown to recruit Histone Deacetylase 1 (HDAC1) to target gene promoters [2]. During embryogenesis, TBX2 is fundamental to the regulation of cell fate decisions, cell migration, and morphogenesis in a variety of organs including the limbs, heart, kidney, nervous system, and eyes [17], [18], [19], [20], [21], [22] – albeit through mechanisms that remain poorly understood. TBX2 is also prominently expressed in the embryonic mammary glands with a restricted expression in breast mesenchymal cells, which give rise to the stroma [23], [24]. Although early embryonic lethality of *Tbx2* knockout mice, due to severe heart defects, has precluded analysis of TBX2 function in mammopoiesis [18], adult heterozygous *Tbx2* mouse mutants exhibit mild mammary gland branching defects, suggesting that TBX2 may be required for normal mammary gland morphogenesis [24].

TBX2 is also implicated in cell cycle regulation [9], [25], whereby overexpression of TBX2 in different *in vivo* model and cell culture systems has shown to both promote [9], [10], [21], [26], as well as attenuate cell proliferation [27], [28]. Inappropriate activation of TBX2 in cancer is thought to contribute to early tumor progression by its ability to over-ride senescence and therefore maintain tumor growth [9]. Senescence is a permanent G1 growth arrest induced by DNA damage or oncogenic insult that represents a failsafe mechanism against cancer [29]. TBX2 has been shown to suppress senescence through both p53-dependent [2], [9], [15], [30] and p53–independent mechanisms [10], [26]. Consequently, TBX2 can cooperate with transforming oncogenes (c-Myc, Ras) or the loss of tumor suppressor genes (p53, Rb) in cellular transformation [9], [31], [32]. Moreover, overexpression of TBX2 in human lung and skin cancer models, although inhibitory to cell growth, has been shown to promote the resistance of tumor cells to the anti-cancer drug cisplatin [28]. Whilst the anti-senescence activity of TBX2 has been extensively studied, it has remained unclear whether TBX2 can also contribute to tumor invasion, as the clinical association of *TBX2* gene amplification with invasive epidermal tumors would suggest.

There is increasing evidence that aberrant activation of the embryonic morphogenetic program, termed the epithelial-mesenchymal transition (EMT), is crucially involved in tumor cell invasion [33]. During EMT, adherent epithelial cells lose polarity, undergo a major reorganization of the cytoskeleton and acquire a fibroblastic (mesenchymal), highly motile phenotype [34]. EMT is potently activated by TGFß [35], and at the transcriptional level by a growing list of embryonic transcription factors (TFs) [33]. These include the *E-cadherin* repressing Zinc finger proteins Snail (SNAI1) and Slug (SNAI2) [36], [37], ZEB1 (∂EF1) and ZEB2 (SIP1) [38], [39]; the basic helix-loop-helix proteins TWIST1/2 [40], [41], [42]; the homeodomain proteins Goosecoid, LBX1 and SIX1 [43], [44], [45]; and the winged-forkhead transcription factor FOXC2 [46]. Virtually all of these TFs have also been implicated as drivers of oncogenic EMT and breast cancer metastasis [47], [48], [49], [50]. EMT is increasingly viewed as a significant clinical problem in cancer, as EMT is thought to promote an aggressive cancer stem cell phenotype [51], [52], therapy resistance [53], and tumor recurrence [54], thereby contributing to poor disease outcome. Thus, there is an urgent need for the identification and characterization of the genes involved in this process.

Through ectopic expression of TBX2 in normal mammary epithelial cells and RNAi-mediated silencing of endogenous TBX2 overexpression in malignant human breast carcinoma cell lines, we demonstrate that TBX2 acts as a strong cell-autonomous inducer of EMT. We found that TBX2 directly represses *E-cadherin* transcription and promotes malignant tumor progression by imparting an aggressive mesenchymal tumor phenotype. These findings, together with a significant correlation between high *TBX2* expression levels in primary tumors and reduced metastasis-free survival of breast cancer patients, suggest that TBX2 may be an attractive new target for anti-metastatic cancer therapies.

## Results

### TBX2 Efficiently Induces EMT in Mammary Epithelial Cells

**Figure 1:**
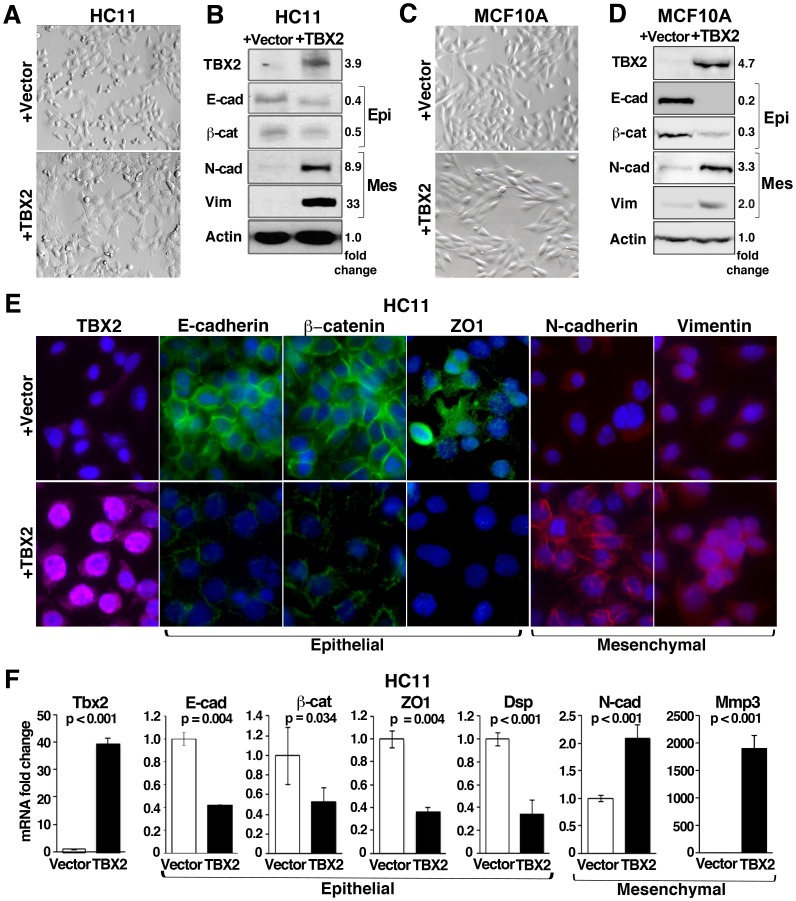
TBX2 induces epithelial-mesenchymal transition (EMT) in breast epithelial cells. (A) Bright field images (40x magnification) of murine HC11 mammary epithelial cells stably transfected with pCDNA3 (+vector) or pCDNA3-TBX2 (+TBX2) expression plasmids showing morphological changes in TBX2-expressing HC11. (B) Western blot analysis using whole cell lysates from HC11+vector and HC11+TBX2 cells shows TBX2-induced downregulation of epithelial (Epi) and upregulation of mesenchymal (Mes) marker proteins. E-cad  =  E-cadherin; ß-cat  =  ß-catenin; N-cad  =  N-cadherin; Vim  =  Vimentin. Actin (ß-actin) was used as loading control. Fold changes in protein levels quantified by densitometry and normalized to Actin values are shown. (C) Bright field images (40X magnification) of human MCF10A mammary epithelial cells stably expressing pCDNA3 or pCDNA3-TBX2 reveal mesenchymal transformation of MCF10A+TBX2 cells. (D) Western Blot analysis shows that ectopic expression of TBX2 in MCF10A cells prompts a switch of EMT marker expression. (E) Immunofluorescence analysis of TBX2 (red) and EMT marker expression (40X magnification) shows loss of membrane-associated expression of epithelial (green) with a reciprocal gain of mesenchymal (red) marker expression in HC11+TBX2 cells as compared to HC11+vector control cells. Nuclei were stained with Hoechst 33258 (blue). (F) qPCR analysis comparing *Tbx2* and EMT marker expression in HC11+vector and HC11+TBX2 cells. E-cad  =  *E-cadherin*; ß-cat  =  *ß-catenin*; ZO1 =  *zona occludens 1;* Dsp  =  *Desmoplakin*; N-cad  =  *N-cadherin*; Mmp3 =  *matrix metalloprotease 3*. Values were normalized to *Gapdh*. Fold changes as compared to vector control cells are shown. Error bars represent the mean ± SEM (n = 3; Student *t*-test). *P* values are indicated.

We were intrigued by previous observations that during mouse embryonic development, *Tbx2* is exclusively expressed in mesenchymal cells surrounding the mammary epithelial anlagen [23], [24], suggesting it may regulate mesenchymal cell specification in the breast. Yet, studies examining TBX2 expression in a small number of human breast cancers have reported TBX2 mRNA and protein overexpression primarily in the epithelial compartment of tumors with little or no expression in stromal cells [7], [55]. We therefore hypothesized that the apparent misexpression of TBX2 in breast epithelial cells during carcinogenesis may confer mesenchymal properties to these cells. To test this hypothesis, TBX2 was stably introduced into murine HC11 and human MCF10A cells, two spontaneously immortalized but otherwise normal mammary epithelial cell lines, which we found lack endogenous TBX2 expression (Figure 1B, 1D and Figure S3A, S3B). To avoid clonal selection bias, several individual polyclonal cell cultures expressing pCDNA3-TBX2 plasmid (+TBX2) or pCDNA3 vector (+vector) alone were established. Each of the TBX2-expressing HC11 and MCF10A cell derivatives (n = 3 per line) showed a dramatic change in cell morphology from the earliest passages onwards (Figure 1A, 1C). While vector-transfected HC11 or MCF10A cells had a typical epithelial cell structure, HC11+TBX2 cells were abnormally enlarged and stretched out with lamelopodia-like migratory protrusions (Figure 1A). In addition, MCF10A+TBX2 cells clearly displayed a spindle-shaped, fibroblastoid, and scattered morphology (Figure 1C). Analysis of EMT marker protein expression by Western Blot and immunofluorescence revealed that the TBX2-induced morphologic changes were due to EMT (Figure 1B, 1D, and 1E). Protein levels of the epithelial adherence junction proteins E-cadherin and ß-catenin were decreased in HC11+TBX2 and MCF10A+TBX2 cells, whereas mesenchymal markers, Vimentin and N-cadherin, were markedly upregulated as compared to the respective vector control cells (Figure 1B, 1D, and 1E). Furthermore, whereas in confluent HC11+vector control cells, E-cadherin, ß-catenin, and the tight junction protein ZO1 were detected primarily at cell-to-cell junctions, TBX2-expressing HC11 cells at the same cell density exhibited a reduced and disrupted immunostaining for these epithelial cell adhesion molecules at the cell periphery (Figure 1E). A breakdown of epithelial adhesion complexes was further evident by a significant reduction in mRNA levels for *E-cadherin*, *ß-catenin*, *ZO1*, and the desmosomal component *Desmoplakin* by 50–70% respectively in TBX2-overexpressing HC11 cells as determined by quantitative realtime PCR (qPCR) (Figure 1F). Of mesenchymal markers analyzed, *N-cadherin,* and the extracellular matrix metalloproteinase *Mmp3* were most significantly upregulated in these cells (Figure 1F). A similar switch from epithelial to mesenchymal marker gene expression was also evident in MCF10A+TBX2 cells (Figure S1A, S1B). Thus, TBX2 efficiently induced morphologic and molecular changes characteristic of EMT in mammary epithelial cells.

### TBX2 Promotes Mammary Epithelial Cell Motility and Invasiveness

**Figure 2:**
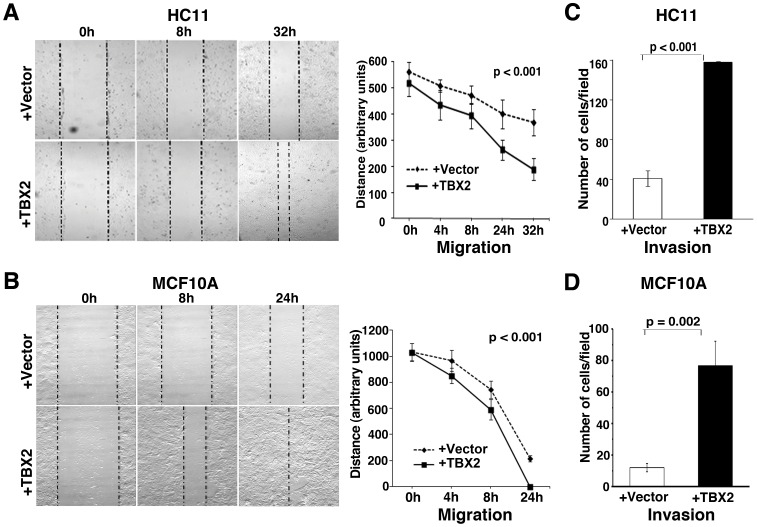
TBX2 promotes migration and invasion of mammary epithelial cells. (A, B) *In vitro* ‘scratch’ assays (see Methods) monitoring the migration of (A) murine HC11 and (B) human MCF10A cells stably expressing pCDNA3 vector (+vector) or pCDNA3-TBX2 (+TBX2) over a period of 24–32 hours (h). Representative bright field images of cells (10x magnification) are shown in the left panel. Right panel: statistical evaluation of the distance between the two borders (dotted lines; left panels) at different time points after ‘scratch’ (n = 3; ANOVA test). (C, D) Transwell matrigel invasion assays show a significantly increased ability of TBX2-expressing HC11 (C) and MCF10A cells (D) to invade through a matrigel layer (n = 3, Student’s *t*-test). The mean ± S.D. is shown. P values are indicated.

We next tested whether ectopic expression of TBX2 promotes any behavioral changes associated with EMT, such as increased motility and gain of invasiveness. Parental and vector-expressing HC11 or MCF10A cells have a low propensity to migrate and invade extracellular matrix (Figure 2). However, both TBX2-overexpressing HC11 and MCF10A cells exhibited a significant increase in cell motility in “*in vitro* scratch” assays, which was visible as early as 4–8 hours after an experimentally induced wound and became more pronounced between 8–32 hours, leading to a complete wound closure by MCF10A+TBX2 cells at 24 hours (Figure 2A, 2B). The increased movement of TBX2-expressing mammary epithelial cells was not due to increased proliferation since the assay was done in low serum-containing medium (see Methods), in which these cells were growth-retarded (data not shown). To examine the role of TBX2 in the regulation of cell invasiveness, we performed Transwell matrigel invasion assays (Figure 2C, 2D). Both TBX2-expressing HC11 and MCF10A cells, showed an approximately four-fold increase in their abilities to invade through the matrigel layer towards serum-containing media (Figure 2C, 2D). Thus, TBX2 plays a central role in the acquisition of cell motility and invasiveness of breast epithelial cells through induction of EMT.

### TBX2 is Induced during TGFß-mediated EMT of Primary Human Mammary Epithelial Cells

**Figure 3:**
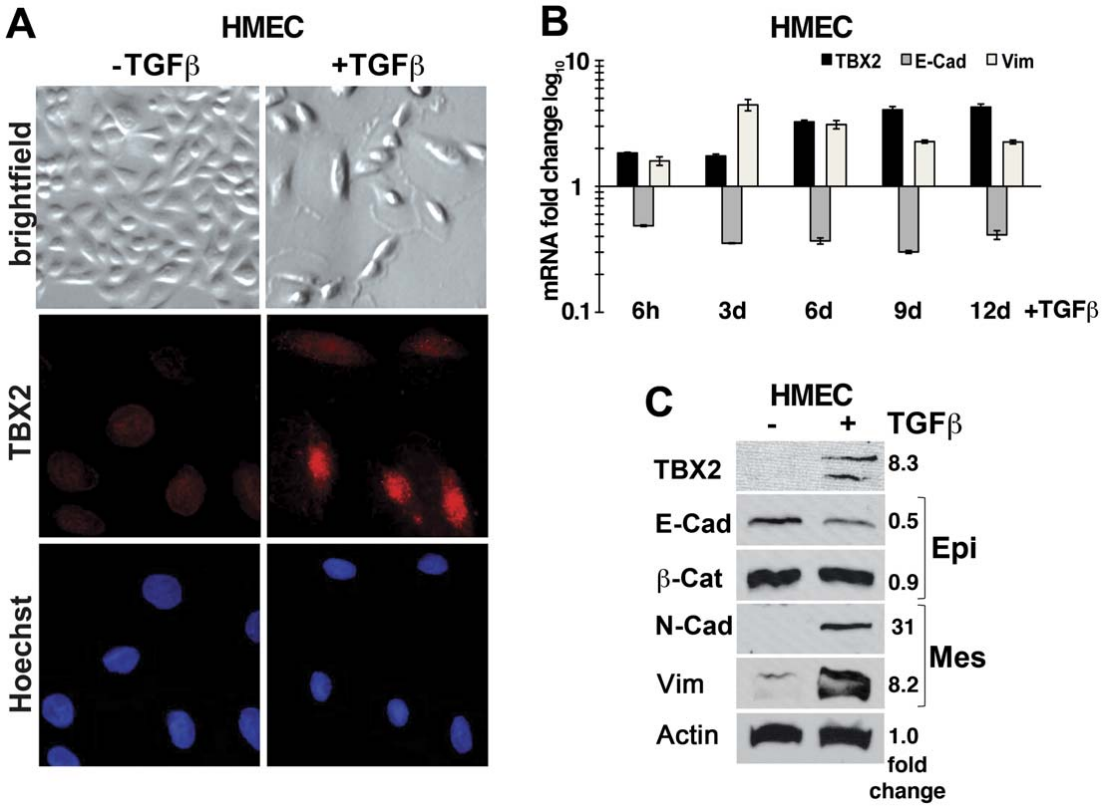
Endogenous TBX2 is induced during TGFß-mediated EMT of primary human mammary epithelial cells (HMEC). (A) Bright field (40x magnification) and immunofluorescence images (63X magnification) of primary HMEC treated with 5 ng/ml TGFß1 (+TGFß) for 12 days as compared to untreated control cells (-TGFß). TGFß induces EMT-like morphological changes and nuclear expression of TBX2 (red). Nuclei were stained with Hoechst 33258 (blue). (B) qPCR analysis shows a time course analysis of *TBX2* mRNA induction in TGFß–treated HMEC in comparison to changes in epithelial *E-cadherin* (E-cad) and mesenchymal *Vimentin* (Vim) expression. Values were normalized to *GAPDH* mRNA and represent fold changes as compared to control untreated HMEC at the indicated time points. Error bars represent SEM of each sample in triplicates. (C) Western blot analysis of TBX2 and EMT marker expression in untreated HMEC (−) and in HMEC treated with TGFß for 12 days. E-cad  =  E-cadherin; ß-cat  =  ß-catenin; Vim  =  Vimentin; N-cad  =  N-cadherin; Epi =  epithelial; Mes  =  mesenchymal markers. Actin  =  ß-actin was used as loading control. Densitometric quantification of fold changes in protein levels normalized to Actin values is shown.

We further examined expression of endogenous TBX2 in a cellular model of inducible EMT. We have previously shown that treatment of primary finite-lifespan human mammary epithelial cells (HMEC) with the cytokine TGFß efficiently induces EMT, as well as a hierarchy of known EMT-associated transcription factors [56]. While TBX2 was not expressed in untreated HMEC (Figure 3A and 3C), both TBX2 mRNA and protein were upregulated upon EMT induction by TGFß coinciding with a decrease in epithelial and acquisition of mesenchymal marker expression (Figure 3A–3C). A significant induction of *TBX2* mRNA expression occurred as early as 6 hours (∼1.8 fold; p<0.001) upon TGFß stimulation and further increased to 3.3–4.3 fold by 9–12 days (p<0.001) (Figure 3B). Notably, the rapid increase of *TBX2* mRNA levels, accompanied by a reduction in epithelial *E-cadherin* and an increase in mesenchymal *Vimentin* mRNA expression (Figure 3B), occurred well before any morphological changes of EMT became visible at 3 days of TGFß treatment [56]. Moreover, TBX2 protein specifically localized to the nucleus of TGFß-treated HMEC (Figure 3A), where it is thought to be active as a transcription factor. These data indicate that TBX2 is also part of the endogenous EMT program of primary HMEC and further implicate TBX2 in EMT induction of breast epithelial cells.

### TBX2 Expression in Human Breast Tumors Correlates with EMT Features and Increased Disease Recurrence

EMT and increased invasiveness are key features of epithelial tumor cells as they progress into malignant metastatic cancer cells [33]. We therefore asked whether the EMT-inducing and pro-invasive abilities of TBX2 that we observed in normal breast epithelial cells, could also play a role in human breast cancer. Since little is known about TBX2 expression in human breast tumors, we performed a comprehensive meta-analysis of *TBX2* expression using an integrated gene expression database that encompassed 1107 primary human breast tumors from six published datasets [57]. We found that *TBX2* was variably expressed across the different molecular subtypes of breast cancer [58] (Figure S2A), largely independent of estrogen receptor (ER) status and tumor grade (Figure S2B, S2C), with the highest expression levels among the rare, aggressive ‘claudin-low’ subtype of breast cancer and lowest in basal tumors (Figure S2A). Interestingly, the ‘claudin-low’ subtype of breast tumors has recently been shown to be enriched for EMT features [59], [60]. Another dataset showed that *TBX2* was higher in poor prognosis metaplastic breast cancers (Figure S2D), which like the ‘claudin-low’ group exhibit an EMT gene signature and are highly metastatic [60], [61]. Expression of TBX2 was not significantly higher in the ‘claudin-low’ tumors of this dataset, but the numbers of tumors (n = 13) were much lower than in the in the ‘six study’ analysis (n = 34) [57].

A similar tumor subtype distribution of TBX2 overexpression was observed in a panel of a total of 20 human breast carcinoma cell lines using Western blot and qPCR analyses (Figures S3 and S4). We confirmed endogenous TBX2 overexpression in three ER-positive luminal tumor lines (MCF7, MDA-MB361, and BT-474) (Figure S3A–C) that have previously been shown to harbor *TBX2* gene amplifications [6], [9], [10]. Moreover, the ER-negative basal breast carcinoma cell line SUM52 [62] displayed abundant TBX2 protein and mRNA expression, as well as modest gene amplification levels (Figure S3A–3C), consistent with previous Fluorescence *In Situ* Hybridization (FISH) data [6]. In addition, we identified TBX2 overexpression in two metaplastic tumor-derived breast carcinoma cell lines, MDA-MB-435 and MDA-MB-157 [63], [64], which we found to express higher median levels of *TBX2* than most other breast tumors (Figure S2D). Both of these cell lines exhibit mesenchymal gene signatures [64], and have an increased invasive, metastatic potential [63], [65]. Comparative genomic hybridization array (aCGH) analysis showed no significant increases in *TBX2* gene copy numbers in MDA-MB-435 and MDA-MB-157 (Figure S3C), suggesting that overexpression of TBX2 in these tumor cell lines is not due to gene amplification. *TBX2* gene amplification has previously been reported in aggressive *BRCA1*-related breast cancers [6], [7]. However none of the four basal-subtype ER-negative *BRCA1*-deficient breast carcinoma cell lines (HCC1937, MDA-MB-436, SUM149, and SUM1315) we studied expressed TBX2 protein at detectable levels or exhibited *TBX2* gene amplification (Figure S4A, S4B). Furthermore, TBX2 was not expressed in any normal-derived human breast epithelial cell line (Figures S3 and 1B, 1D).

**Figure 4:**
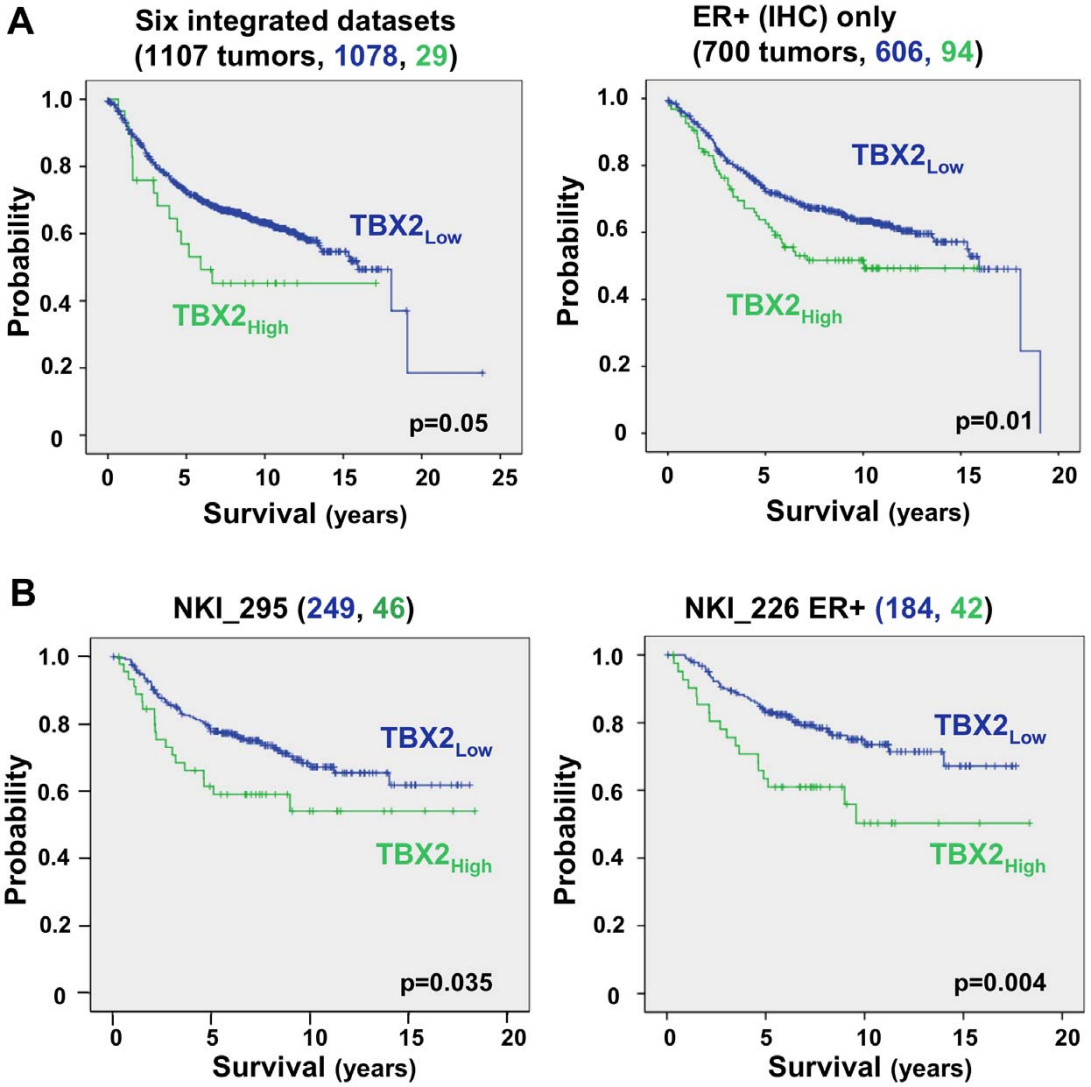
*TBX2* overexpression in primary human breast tumors is correlated with reduced metastasis-free survival. Kaplan Meier analysis demonstrates that *TBX2* mRNA overexpression is associated with shortened recurrence-free survival (A) in a meta-analysis of six combined published microarray datasets comprising 1107 primary human breast tumors [57], and (B) in an NKI study of 295 women with early-stage invasive breast carcinomas [66]. In both of these datasets, the optimal cut-point value of *TBX2* expression was used to divide the samples into high (above median; green) and low (below median; blue) *TBX2* expression. A batch correction was performed on the six-study set (see Methods). High *TBX2* expression is particularly associated with poor prognosis in estrogen receptor-positive (ER+) breast tumors (right panels). ER status was determined by immunohistochemistry (IHC). P values are indicated.

The prognostic significance of TBX2 in human breast cancer was examined next by meta-analysis. High *TBX2* transcript levels were found to be significantly associated with a shorter time to recurrence-free survival (Figure 4A, 4B). This was more significant for ER-positive tumors and similar results were seen in the completely independent 295-sample NKI dataset [66] (Figure 4A, 4B). Overall, these results are compatible with the notion that TBX2 is activated in certain primary breast cancers correlating with an EMT signature and reduced metastasis-free survival.

### Silencing of TBX2 Leads to Mesenchymal-epithelial-transition and Impedes the Invasiveness of Human Breast Cancer Cells

**Figure 5:**
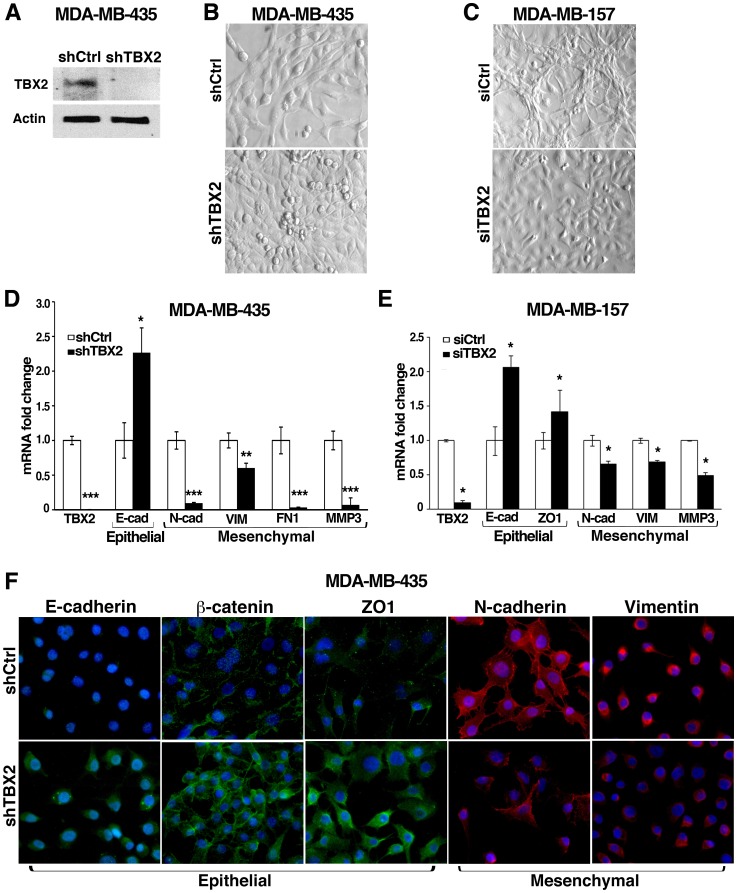
TBX2 imparts a mesenchymal phenotype on human breast cancer cells. (A) Western blot analysis shows efficient TBX2 knockdown in human MDA-MBA-435 tumor cells stably expressing TBX2-specific shRNA (shTBX2) as compared to cells expressing non-target shRNA (shCtrl). Actin was used as loading control. (B) Depletion of TBX2 in MDA-MB-435 tumor cells leads to a loss of the mesenchymal morphology characteristic for this breast carcinoma cell line. Representative images of high-density cell cultures are shown (40X magnification). (C) Inhibition of TBX2 in human MDA-MB-157 breast carcinoma cells through transient transfection with TBX2-targeted siRNAs (siTBX2) induces a ‘cobblestone’-like epithelial cell morphology. In contrast, MDA-MB-157 cells transiently transfected with scrambled siRNA control (siCtrl) exhibit a profound ‘spindle-like’ mesenchymal phenotype. Representative images of high-density cultures (40x magnification) of tumor cells three days post siRNA transfection are shown. (D, E) qPCR analysis of *TBX2* and EMT marker expression in (D) MDA-MB-435 cells expressing shCtrl or shTBX2, and in (E) MDA-MB-157 cells 3 days post transfection with siCtrl or siTBX2. TBX2 knockdown leads to an upregulation of epithelial adhesion and tight junction genes (E-cad  =  *E-cadherin*; ZO1 =  *zona occludens 1*), whereas it results in loss of mesenchymal marker expression: N-cad  =  *N-cadherin*; VIM  =  *Vimentin*; FN1 =  *Fibronectin*, and MMP3 =  *matrix metalloprotease 3*. Values were normalized to *GAPDH* and fold changes compared to the respective control groups are shown. Data represent the mean ± SEM (n = 3; Student *t*-test); p-values: *p<0.05; **p = 0.003; ***p<0.001. (F) Immunofluorescence analysis (40X magnification) confirms the re-expression of epithelial (green) E-cadherin, ß-catenin, and ZO1, and the loss and/or mislocalization of mesenchymal (red) markers (N-cadherin, Vimentin) in TBX2-depleted MDA-MB-435 cells. Nuclei were stained with Hoechst 33258 (blue).

**Figure 6:**
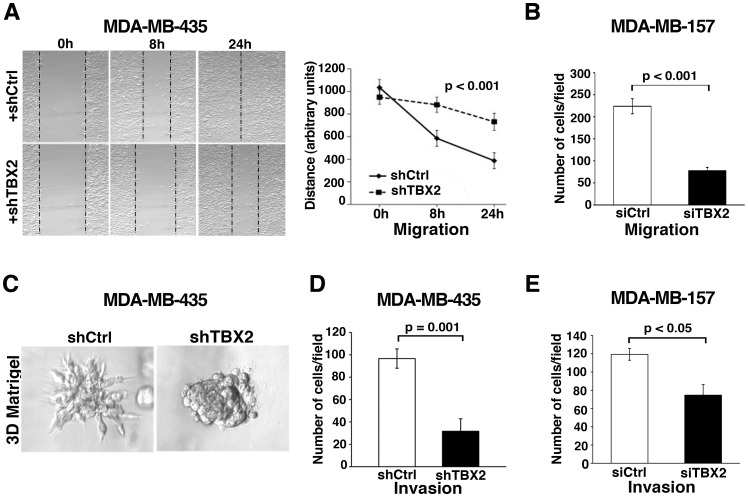
Knockdown of TBX2 in metastatic human breast cancer cell lines abrogates tumor cell invasion. (A, B) Inhibition of TBX2 significantly reduces cell motility rates of (A) MDA-MB-435 tumor cells in *in vitro* scratch assays, and (B) of MDA-MB-157 breast cancer cells in Transwell migration assays (see Methods). Data represent the mean ± S.D. (n = 3; ANOVA test). (**C**) MDA-MB-435 cells stably expressing TBX2-specific shRNA (+shTBX2) grow as non-invasive spheres in three-dimensional (3D) Matrigel, whereas MDA-MB-435 control tumor cells expressing non-target shRNA (+shCtrl) grow as spheroids that invade the surrounding extracellular matrix. (D, E) Transwell matrigel assays showing that knockdown of TBX2 significantly reduces invasion rates of (**D**) MDA-MB-435, and (E) MDA-MB-157 breast tumor cells. Data represent mean ± S.D. (n = 3; Student’s *t*-test). P values are indicated.

To elucidate the potential role of TBX2 in malignant tumor progression, we employed RNA interference strategies to inhibit TBX2 in the metastatic breast carcinoma cell lines MDA-MB-435 and MDA-MB-157 (Figures 5 and 6). Efficient TBX2 knockdown was achieved in MDA-MB-435 tumor cells by stable transduction with lentiviruses expressing TBX2-specific shRNA (shTBX2), and in MDA-MB-157 cells by transient transfection with TBX2-specific siRNAs (siTBX2) (Figure 5A, 5D and 5E). Notably, whereas MDA-MB-435 cells transduced with control non-target shRNA (shCtrl), or MDA-MB-157 cells transiently transfected with control scrambled siRNAs (siCtrl), had a fibroblastoid ‘spindle-like’ appearance similar to the respective parental cell lines, TBX2-depleted MDA-MB-435 and MDA-MBA-157 tumor cells displayed a ‘cobblestone-like” epithelial morphology (Figure 5B, 5C). Concordant with this phenotype, mRNA expression of epithelial *E-cadherin* and *ZO1* was enhanced, whereas transcription of mesenchymal genes (*N-cadherin, Vimentin, Fibronectin, MMP3*) was reduced in TBX2-depleted MDA-MB-435 and MDA-MB-157 breast cancer cells (Figure 5D, 5E). Immunofluorescence analysis further confirmed that TBX2 inhibition resulted in the re-expression of E-cadherin protein in MDA-MB-435 cells, which are normally devoid of this epithelial marker (Figure 5F) [35]. Moreover, epithelial ß-catenin and ZO1 not only were increased in levels, but also properly localized to the cell membrane in TBX2-depleted MDA-MB-435 tumor cells (Figure 5F). In contrast, mesenchymal markers (N-cadherin, Vimentin) were drastically reduced and, for N-cadherin, mislocalized to the cytoplasm in MDA-MB-435-TBX2 knockdown cells (Figure 5F).

Inhibition of TBX2 further led to reduced tumor cell migration in both MDA-MB-435 and MDA-MB-157 breast carcinoma cell lines (Figure 6A, 6B). Moreover, tumor cell invasion rates in Transwell matrigel invasion assays were markedly decreased (Figure 6D, 6E). Additionally, whereas control non-target shRNA expressing MDA-MB-435 tumor cells formed spheroids with extensive protrusions in three-dimensional (3D) Matrigel cultures, reflective of their invasive nature, knockdown of TBX2 resulted in the formation of round, non-invasive spheres (Figure 6C). Thus, loss of TBX2 in malignant breast carcinoma cells abolished tumor cell invasion and lead to the restitution of a more differentiated epithelial phenotype. All experiments were done with at least two independent cell clones from each stably shRNA transfected cell line and three independent polyclonal cultures of cell lines transiently transfected with siRNAs.

### TBX2 is Crucial for Metastasis of Breast Carcinoma Cells

**Figure 7:**
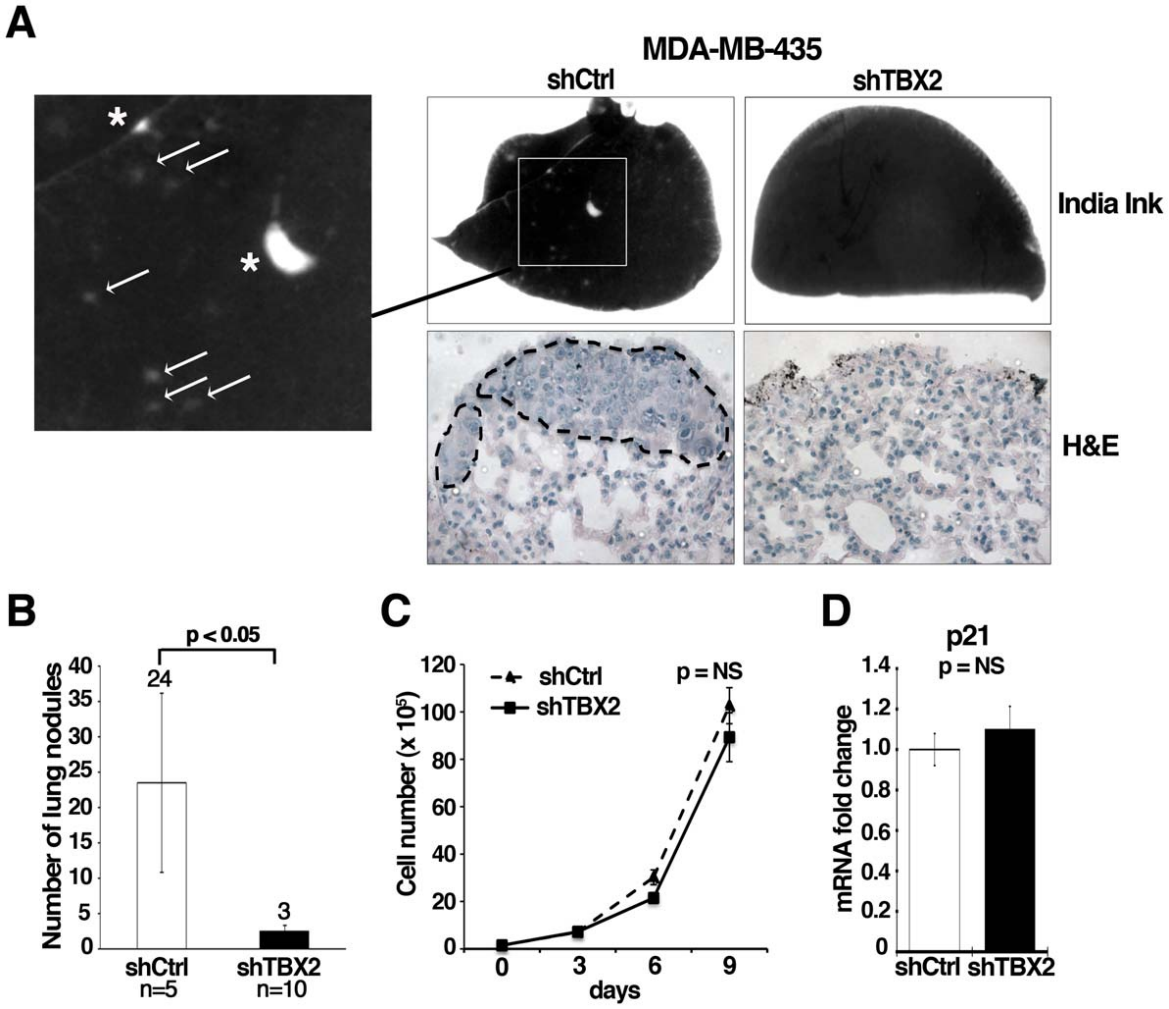
Knockdown of TBX2 reduces pulmonary metastasis of human MDA-MB-435 breast carcinoma cells. (A) Representative images of lungs harvested from athymic *nu/nu* Nude mice forty days after tail vein injection with MDA-MB-435 tumor cell clones expressing either control non-target shRNA (shCtrl) or TBX2-specific shRNA (shTBX2). Top panel: India ink staining of lungs shows the absence of surface lung metastases in mice injected with shTBX2-expressing MDA-MBA-435 tumor cells (Magnification 7x). Only the control group produced macroscopic lung nodules (asterixes) and an elevated number of micrometastases (white arrows). Bottom panel: H&E stained paraffin-sections of representative lungs from each study group (Magnification: 40X). Dotted lines highlight lung metastases in the control group. (B) Quantification of total lung metastasis burden in the same sets of mice as in (A). Average numbers of lung surface metastases are shown; white column  =  mean of 5 control mice analyzed: black column  =  mean of 10 mice injected with two MDA-MB435-shTBX2 tumor cell clones. Data represent the mean ± S.D. (n≥5; Student *t*-test). (C) Inhibition of TBX2 does not significantly affect cell proliferation of MDA-MB-435 tumor cells. Equal numbers of control non-target shRNA and shTBX2-expressing cells were grown under sub-confluent conditions and counted every 3 days over a 9-day period. Error bars represent the mean ± S.D. (n = 3; Student *t*-test). (D) qPCR showing that stable knockdown of TBX2 does not significantly alter *p21* mRNA expression levels in MDA-MB-435 tumor cells. Values were normalized to *GAPDH* and fold changes compared to the shRNA control group are shown. Error bars represent the mean ± SEM (n = 3; Student *t-*test). NS  =  not significant.

To further investigate how aberrant overexpression of TBX2 contributes to malignant tumor progression, we analyzed the metastatic potential of TBX2-depleted MDA-MB-435 tumor cells. MDA-MB-435 cells expressing either shCtrl or shTBX2 were injected into the tail veins of immunocompromised *nu/nu* Nude mice. Lung colonization was assessed 40 days post tumor cell transplantation. Functional inhibition of TBX2 in MDA-MB-435 (two independent clones: shTBX2 C2 and shTBX2 C4) led to a strong reduction in the number of lung nodules and micrometastases as compared to MDA-MB-435+shCtrl cells (Figure 7A, 7B). Growth rates were not significantly reduced for shTBX2 expressing MDA-MB-435 tumor cells in culture (Figure 7C). Moreover, mRNA expression of the cell cycle inhibitor p21^CIP1/WAF1^, which is a direct transcriptional target of TBX2 in senescence bypass [2], [30], was not significantly altered in TBX2 knockdown cells (Figure 7D), nor did these cells exhibit any signs of senescence (data not shown). These data suggest that TBX2 is crucial for the formation of metastases independent of effects on tumor cell growth. Taken together, our TBX2 inhibition studies in malignant breast carcinoma cell lines reinforce the notion that TBX2 promotes malignant tumor progression by imparting a highly invasive mesenchymal phenotype on breast epithelial tumor cells.

### TBX2 Represses *E-cadherin* Transcription

Loss of *E-cadherin* is an important hallmark of EMT, directly contributing to transformation and metastatic tumor progression [67], [68]. Studies in melanoma cells have raised the possibility that TBX2 may be implicated in *E-cadherin* regulation, but failed to detect a requirement of TBX2 for *E-cadherin* expression *in vivo* [69]. Since we found overexpression of TBX2 to consistently reduce endogenous *E-cadherin* levels in normal mammary epithelial cells, and, conversely, inhibition of TBX2 to lead to enhanced *E-cadherin* mRNA expression in metastatic breast cancer cell lines (Table 1, Figures 1F and 5D, 5E), we revisited the question whether TBX2 could directly repress *E-cadherin* at the promoter level.

**Table 1:** Overexpression of TBX2 in normal breast epithelial cell lines results in reduced expression of *E-cadherin* and knockdown of TBX2 in breast carcinoma cell lines leads to an increase of *E-cadherin* expression.

Cell line	Percentage relative mRNA expression (*TBX2*)	Percentage relative mRNA expression(*E-cadherin*)
HC11+vector	100	100
HC11+TBX2	3882	30
MCF10A+vector	100	100
MCF10A+TBX2	305	64
MDA-MB-435+shCtrl	100	100
MDA-MB-435+shTBX2	3	226
MDA-MB-157+siCtrl	100	100
MDA-MB-157+siTBX2	14	207

**Figure 8:**
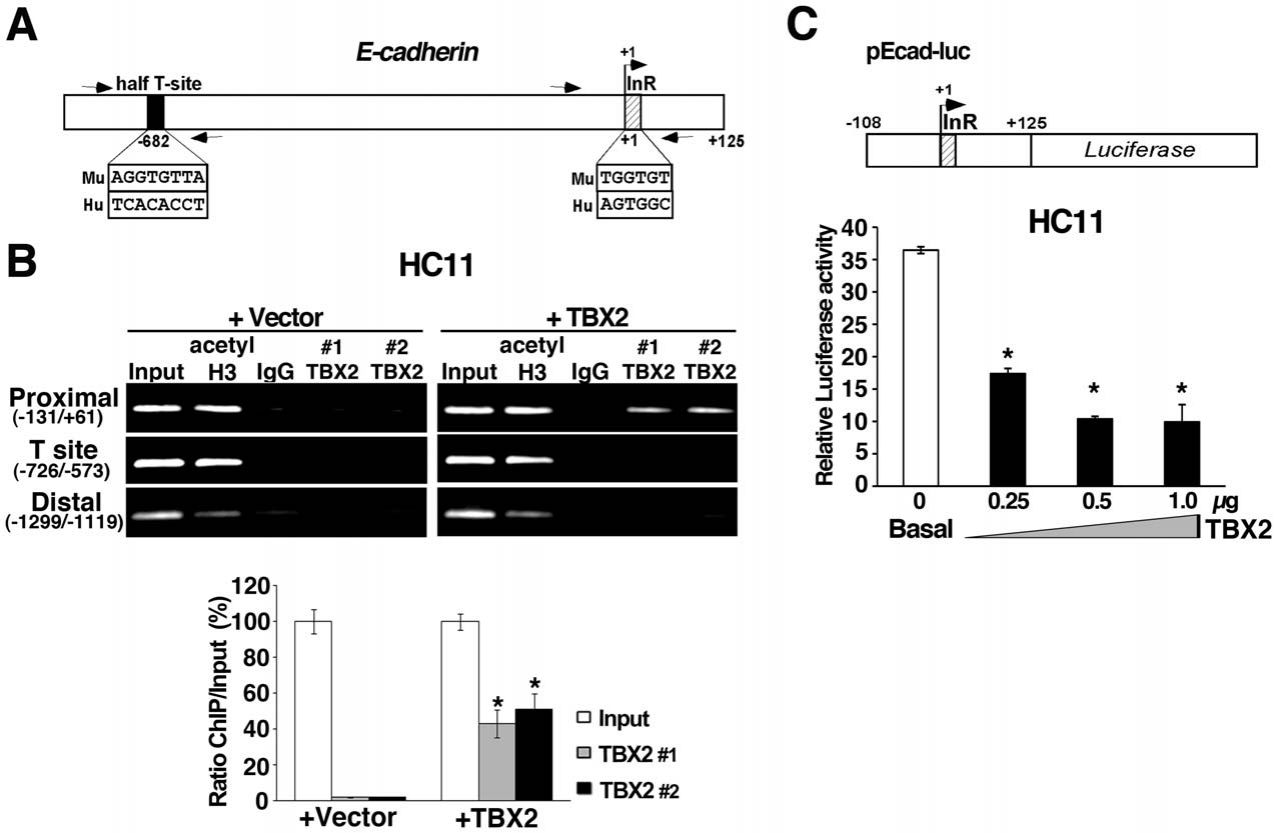
TBX2 bind to the *E-cadherin* promoter *in vivo and* represses *E-cadherin* transcription. (A) Schematic of the *E-cadherin/CDH1* promoter depicting the location of potential TBX2 binding sites [10]: half T-site (black box) and InR  =  Initiator element (hatched box), and of ChIP primers used in (B). (B) ChIP analysis shows *in vivo* binding of exogenous TBX2 to the most proximal region of the endogenous *E-cadherin* gene promoter in HC11 mammary epithelial cells. DNA derived from sheared chromatin fragments from HC11+vector and HC11+TBX2 was immunoprecipitated with two antibodies specific to TBX2 (#1 =  Millipore AB4147; #2 =  SC-17880x), an antibody specific to acetyl Histone 3, or normal rabbit IgG and quantified by semi-quantitative PCR. As a control, <1% of input chromatin was used in the PCR analysis. The bar graph on the bottom panel shows a quantification of the TBX2-specific ChIPs for the proximal (−131/+61) *E-cadherin* promoter as a function of the percentage of input chromatin. (C) Transient reporter assays of HC11 cells transiently co-transfected with a human *E-Cadherin* promoter (−108 to +125) luciferase reporter construct (pEcad-luc) in combination with pCDNA3 vector (basal) or increasing concentrations of pCDNA3-TBX2 (+TBX2), as indicated. One representative experiment of n = 3 biological replicates is shown; P-value: *p<0.05 (triplicate samples; Student *t*-test).

Chromatin immunoprecipitation (ChIP) analysis was performed to determine direct *in vivo* binding of TBX2 to the endogenous *E-cadherin* gene in mammary epithelial cells (Figure 8A, 8B). We used three different primer sets; one covering the most proximal promoter region of *E-cadherin,* including the initiator element (InR: TGGTGT in mouse and AGTGGC in human at +1 to +6 each), which has previously been shown to be bound by a recombinant TBX2 DNA binding domain *in vitro* [69]; one set spanning a conserved half T-box factor binding site (Half T-site: AGGTGTTA at −682 in mouse and TCACACCT at −645 in human) [69]; and one primer set in a distal region (−1299/−1119) devoid of potential TBX2 binding sites (Figure 8A). PCR amplification of these genomic sequences using immunoprecipitated chromatin from HC11+vector or HC11+TBX2 cells demonstrated that TBX2 specifically bound to the proximal *E-cadherin* promoter (Figure 8B), which contains the InR element (Figure 8A) that can serve as a putative TBX2-binding site [15], [69].

To test the functional significance of *in vivo* TBX2 occupancy of this *E-cadherin* promoter region, we transiently co-transfected HC11 cells with a luciferase reporter construct (pEcad-luc) containing the proximal human *E-cadherin* promoter (−108 to +125) [70] and with increasing concentrations of pCDNA3-TBX2 expression plasmid (Figure 8C). As compared to empty vector control (basal), co-expression of TBX2 led to a significant (*p<0.05*) 3.7 fold reduction in the activity of this promoter in a dose-dependent manner. Together, these results indicated that TBX2 directly represses transcription of *E-cadherin* by binding to its proximal promoter *in vivo*.

## Discussion

**Figure 9:**
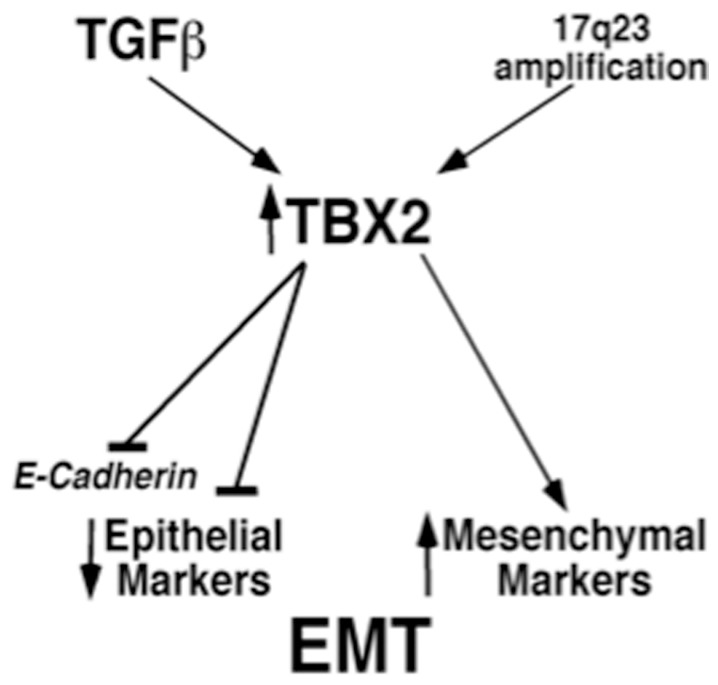
Proposed model for TBX2 regulation of EMT. TGFß signaling, or amplification of human chromosomal region 17q23 lead to aberrant TBX2 expression in differentiated breast epithelial cells, which normally lack TBX2 expression. TBX2 directly represses transcription of the epithelial differentiation marker *E-cadherin* and downregulates other epithelial markers. In contrast, TBX2 induces expression of mesenchymal markers, thereby resulting in EMT and invasion of normal and malignant breast epithelial cells.

In this study, we have identified the embryonic transcriptional repressor and anti-senescence factor TBX2 as a novel potent inducer of EMT that directly represses *E-cadherin* transcription and promotes an aggressive, mesenchymal breast tumor phenotype (Figure 9). Since TBX2 is aberrantly amplified with high prevalence in a number of aggressive human epidermal cancers, or, as we found, can be induced by TGFß (Figure 9), a promoter of metastatic tumor progression [35], these findings are of potential high clinical relevance. Unlike other EMT-inducing TFs [33], TBX2 has not previously been implicated in the cell-autonomous regulation of EMT induction during embryogenesis. Our results therefore uncover a novel paradigm of TBX2 function that may also be relevant for its role in normal development.

During embryogenesis, TBX2 is expressed after gastrulation [11], [23], and among other roles, controls mesenchymal cell specification in the limb buds underlying posterior digit identity [17], specification and morphogenesis of mesoderm-derived cardiomyocytes in the valve forming regions of the heart [18], [71], pro-mesonephros identity during kidney development [19], and cell migration processes during brain and eye morphogenesis [20], [22]. However, the consequences of inappropriate gain-of-function of this morphogenetic TF in epithelial tumor cells during carcinogenesis have been poorly explored. By modeling aberrant gain-of-function of TBX2 in breast cancer through ectopic expression of TBX2 in non-malignant mammary epithelial cell lines (HC11, MCF10A), we demonstrated that TBX2 alone is sufficient to induce EMT and cell invasion.

Since EMT has been implicated in tumor recurrence [53], [54], and we found high levels of *TBX2* expression to be significantly correlated with disease recurrence in a meta-analysis of over 1,000 primary human breast tumors (Figure 4), we further investigated whether the EMT-inducing ability of TBX2 could play a role in malignant tumor progression. Congruent with our TBX2 overexpression results in normal breast epithelial cells, RNAi-mediated silencing of TBX2 in two aggressive mesenchymal human breast carcinoma cell lines with endogenous TBX2 overexpression (MDA-MB-435, MDA-MB-157) led to the restitution of a differentiated epithelial tumor phenotype. This was most evident by re-expression of E-cadherin, ß-catenin, and ZO1, and the concomitant loss of mesenchymal N-cadherin, Vimentin, and Fibronectin expression (Figure 5). Importantly, we found that inhibition of TBX2 also abolished tumor cell migration, invasion and profoundly diminished the capacity of MDA-MB-435 cancer cells to form pulmonary metastases in a xenograft *in vivo* mouse model. These effects appeared to be independent of the anti-senescence activity of TBX2, as cell proliferation and expression of the growth control gene p21^CIP1/WAF1^ were unchanged in MDA-MB-435-shTBX2 cells (Figure 7). The observed pro-invasive activity of TBX2 was likely due to specific induction of MMP3, which unlike other matrix metalloproteases (MMP2, MMP9) was most consistently upregulated by TBX2 in our cellular systems (Figures 1, 4, S1; data not shown). Together, these results suggest that TBX2 is strongly implicated in malignant tumor progression by promoting an aggressive mesenchymal tumor phenotype.

Interestingly, we found that TBX2 is induced by the EMT-promoting cytokine TGFß, which is often excessively produced by invasive breast cancer cells and has been associated with metastatic tumor progression [35]. Because of the rapid kinetics of *TBX2* induction in HMEC upon TGFß treatment, it is tempting to speculate that TGFß signaling controls TBX2 expression at the transcriptional level. This notion is further supported by studies demonstrating that BMPs, which are TGFß homologues, positively regulate *Tbx2* expression during cardiogenesis via functional SMAD binding sites in the *Tbx2* gene promoter [72]. Thus, apart from chromosome 17q23 amplification, TBX2 induction by TGFß may represent a novel mechanism underlying the aberrant overexpression of TBX2 in invasive cancers (Figure 9).

Through ChIP analysis and cell-based promoter-reporter assays, we further demonstrate that TBX2 binds directly to and represses the activity of the proximal *E-cadherin* promoter, indicating that *E-cadherin* is a direct TBX2 target gene. Given that E-cadherin acts as a tumor suppressor, whose loss is causally implicated in EMT and metastatic tumor progression [67], [68], transcriptional repression of *E-cadherin* by TBX2 may provide a possible mechanistic explanation for the observed EMT-inducing and pro-metastatic activities of TBX2 in breast cancer cells. We note that there was little correlation between TBX2 expression and E-cadherin status in established breast cancer cell lines (Figure S3) [90], a finding, which has also been reported for the EMT-inducing transcription factor LBX1 [44]. This could suggest that efficient repression of *E-cadherin* by TBX2 might require cooperation with other transcriptional repressors that may not be present in all TBX2-overexpressing breast tumor cell lines. Moreover, established tumor cell lines do not necessarily reflect the situation in primary breast tumors, in which EMT is histopathologically difficult to detect due to its transient nature [73].

In discrepancy with our results, previous studies have indicated that TBX2 knockdown in MCF7 breast carcinoma and human melanoma cell models did not diminish tumor cell invasion [69], [74] or repress endogenous *E-cadherin* expression [69]. However, TBX3, a close TBX2 homolog with similar oncogenic functions [75] not yet implicated in EMT regulation, exerted these effects in both of those systems [69], [74]. A possible explanation for these conflicting results could be that invasion of the inherently low-invasive MCF7 cell line in the previous study was induced by phorbol esters [74], which might have altered cellular signaling involved in TBX2-mediated EMT induction. Alternatively, there could be cell-type specific differences in the activities of TBX2 and TBX3 that may depend on phosphorylation status or the differential availability of protein partners. TBX2 has been shown to be phosphorylated by external stimuli that alters its cellular localization and, thereby, its activity as a transcription factor [12]. Furthermore, senescence suppression by TBX2 in human cells depends on the physical interaction of TBX2 with tumor suppressive TFs, for example EGR1 in breast cancer cells [10], and PML in fibroblasts [26]. Interestingly, recent studies have indicated that transcriptional repression of the senescence-associated tumor suppressor p14ARF by TBX2 [9], [15], requires the association of TBX2 with NRAGE, a protein that is released from the cell membrane upon EMT induction [76]. To rule out the possibility that TBX3, which is primarily overexpressed in luminal E-cadherin-positive breast cancer lines and estrogen receptor-positive breast tumors [77], [78], contributed to the pro-invasive effects elicited by TBX2, we investigated TBX3 expression in our TBX2-dependent breast epithelial model systems (Figure S5). We found a reciprocal expression of these T-box factors, with TBX3 downregulated in TBX2-expressing HC11 cells but upregulated in TBX2-depleted HC11+TBX2 and MDA-MB-435 tumor cells (Figure S5A, S5B), which is reminiscent of the mutually exclusive expression patterns of TBX2/3 in normal mammary gland tissues [23], [79], Thus, the TBX2-induced EMT phenotypes were not due to a possible interference by TBX3 but rather may reflect the poorly explored role of TBX2 as a mesenchymal and baso-myoepithelial transcription factor in breast development [23], [24]. In contrast, TBX3 functions as a master regulator of mammary epithelial cell fate [80], [81], and in the adult breast is specifically expressed in differentiated luminal breast epithelial cells [79].

Significantly, a role of TBX2 in oncogenic EMT and malignant breast cancer progression is further suggested by our finding that *TBX2* mRNA levels in clinical human breast cancer specimen were highest in rare EMT-enriched tumors of the ‘claudin-low’ and metaplastic breast tumor subtypes. These tumors represent one of the most aggressive and treatment-resistant forms of breast cancer [59], [60], [61]. Moreover, we found high *TBX2* transcript levels to be significantly associated with reduced metastasis-free survival of breast cancer patients, which is in keeping with the correlation of *TBX2* gene amplification data with poor clinical outcome [6], [8]. Since tumor tissues used for expression profiling are subjected to histology and only included if they contain a reasonable percentage of tumor cells it is unlikely that the observed correlations are due to expression of TBX2 in tumor associated stroma. It was perhaps surprising that *TBX2* expression was predictive of poor prognosis but independent of ER status and that basal subtype and high-grade breast tumors had slightly lower average levels of *TBX2* expression (Figure S2). However, these findings are consistent with TBX2 having a similar pattern of gene expression across subtypes as other EMT-inducing TFs, e.g. Twist, ZEB1, ZEB2, and SNAI2 (Slug) [60]. Furthermore, recent clinical population studies have shown that even breast tumors in the lowest risk category (ER+, early stage, small tumor size, node-negativity) and despite adjuvant treatment can have relatively high relapse rates [82], [83]. Thus, TBX2 may prove to have a unique value as a novel prognostic marker.

Collectively, our work suggests that TBX2 is a key driver of malignant tumor progression through induction of EMT and tumor cell invasiveness. Although previous mouse developmental genetic studies have indicated that TBX2 can indirectly promote EMT of endocardial cells during cardiac valve formation through induction of paracrine TGFß2 signaling in surrounding valve-forming myocardium [72], our work is the first to demonstrate that TBX2 can also activate EMT in a cell-autonomous manner. Further experiments are under way to identify the signaling mechanisms, potential interacting partners, and target genes of TBX2 in EMT induction and epithelial tumor invasion. Finally, our discovery that TBX2, an established anti-senescence factor, is a strong inducer of EMT lends further support to the notion that EMT and senescence bypass may rely on some of the same molecular mechanisms [41], [42]. We anticipate our studies to be a starting point for evaluating TBX2 as a new marker for breast cancer diagnosis and potential target for anti-metastatic cancer drug development.

## Methods

### Ethics Statement

All experiments including the use of mice were approved by the University of Miami IACUC (protocol number 10–226, Institutional assurance number for the University of Miami A-3224-01). For the studies we describe, there are no suitable alternative approaches, and care was taken to minimize animal distress.

### Cell Lines

The HC11 mouse mammary epithelial cell line [84] (kindly provided by Dr. Kermit Carraway, University of Miami) was grown in complete growth medium (RPMI containing 10% FBS, 1% penicillin-streptomycin, 1 µg/ml EGF (Invitrogen) and 5 µg/ml insulin (Sigma*-*Aldrich). Human MCF10A mammary epithelial cells were obtained from the American Type Cell Collection (ATCC) and grown in HuMEC medium (Invitrogen). Linearized pCDNA3 or pCDNA3-TBX2 expression plasmids [85] (kindly provided by Dr. Roni Bollag, Medical College of Georgia, Augusta, GA, USA) were introduced into cells by Lipofectamin 2000 transfection (Invitrogen) and stable transfectants were selected in 200–300 µg/ml G418 for 9–11 days. Primary HMEC were from Cambrex Bio Science and cultured in HuMEC medium. TGFß induction experiments using 5 ng/ml of recombinant TGFß1 (R&D Systems) were performed as described [56]. All human breast carcinoma cell lines, except the SUM lines (Asterand), were from ATCC and cultured according to the distributors’ recommendations. Specifically, MDA-MB-435 and MDA-MB-157 cells were grown in DMEM plus 10% FBS and 1% Penicillin-Streptomycin, hereafter referred to as complete growth medium. All cell lines were maintained in a 5% CO_2_-humidified incubator at 37°C.

### Western Blot Analysis

Immunoblotting used 20 µg of total cell extracts and was performed as described [86]. Primary antibodies were to TBX2 1:1,000 (sc-17880x; Santa Cruz Biotechnology), E-cadherin 1∶1,000 (610181; BD Biosciences), ß-catenin 1∶500 (610153; BD), vimentin 1∶2,000 (VIM 13.2; Sigma), N-cadherin 1∶1,000 (610920; BD), or ß-actin 1∶10,000 (AC-15; Sigma). Secondary antibodies were HRP-conjugated anti-goat and anti-mouse IgG (Invitrogen). Protein bands were detected by enhanced chemiluminescence using the Femto Western Blot kit (Pierce). Densitometry to quantify protein expression levels was performed using ImageJ software (NCBI).

### Immunofluorescence

Fluorescence immunocytochemistry on cultured cells was performed as previously described [56]. Primary antibodies were to TBX2 (1∶200; AB4147; Upstate-Millipore), E-cadherin (1∶1,000; BD), ß-catenin (1∶200; BD), ZO1 (1∶500; 61–7300; Zymed), N-cadherin (1∶500; BD), vimentin (1∶500; Sigma), followed by incubation with Alexa Fluor 488- or Alexa Fluor 594-conjugated secondary antibodies (1∶200; Invitrogen). Nuclei were stained with 50 µg/ml Hoechst 33258 (Sigma) in PBS and cells were visualized on a Leitz Axiovert microscope after mounting in Prolong Gold anti-fade reagent (Invitrogen).

### Quantitative Real-Time PCR (qPCR)

qPCR analysis was performed as previously described [56], [86] using SYBR Green PCR Master Mix (New England Biolabs) and a CFX96 Real Time PCR thermocycler (Biorad). Sequences of qPCR primers are shown in Table S1. Triplicate samples were performed and average C_t_ values were normalized to the values of *GAPDH*.

### *In vitro* Scratch Assay

Scratch assays were performed as described [87] with the following modifications. 1×10^6^ cells were plated on a 60 mm dish and cultured in complete growth media 24 h before the assay. When cells reached confluence, a p200 pipette tip was used to scrape a straight line through the cell monolayer. Cells were washed once with growth medium to remove cell debris and to smoothen the edge of the scratch. The culture medium was then replaced with growth medium with a lower FBS content (5% FBS) to minimize cell proliferation during the duration of the assay. Images were taken at different time points after scratch on a Leica DMIL inverted microscope using a Q-Imaging digital camera (Micropublisher) and analyzed using ImageJ software (NCBI). Acquisition of same field images was achieved by making reference points using an ultra fine tip marker.

### Transwell Migration and Matrigel Invasion Assays

Cells (1–2×10^4^) were resuspended in 100 µl of serum-free growth medium and plated into uncoated or Matrigel-coated 8-µm transwell filter inserts (Corning) of 24-well plates in triplicates. For invasion assays, filters were pre-coated with 10 µl of matrigel (BD) diluted 1∶4 in ice-cold serum-free medium and allowed to solidify for 1 h at 37°C before use. The bottom wells contained 500 µl of growth medium with 10% FBS as chemoattractant. After incubation of cells for 16 h for migration or for 48 h for invasion assays, cells on the upper surface of the filter were removed with a cotton swab and cells on the bottom side were fixed in 100% methanol and stained with 1% Toluidine Blue in 1% Borax. Cells were counted in three random fields on microscopic images taken at 10x or 40x magnification.

### RNAi, Lentiviral shRNA Transduction

HC11+TBX2 murine mammary epithelial and human MDA-MB-157 breast tumor cells were transiently transfected with scrambled control or *TBX2* gene-specific Smartpool siRNAs (Dharmacon) at a final concentration of 100 nM using Dharmafect #1 transfection reagent according to manufacturer’s protocol. Changes in EMT marker expression and cell motility/invasion were evaluated 3–4 days post siRNA transfection. For stable TBX2 knockdown, MDA-MB-435 cells were initially transduced with 5 different *TBX2*-specific shRNAs (Mission shRNA lentiviral particles; Sigma, TRCN0000232146-150) or control non-target shRNA (Sigma, SHC002V) at MOI = 5. TBX2 shRNA TRCN0000232147 (5′CCGGTGAGATGCCCAAACGCATGTACTCGAGTACATGCGTTTGGGCATCTCATTTTTG-3′) yielded the highest TBX2 knockdown efficiency in qPCR and WB analysis and was used to generate stable TBX2 knockdown cells. Individual MDA-MB-435 cell clones stably expressing TBX2 shRNA or control shRNA were obtained by selection in 1 µg/ml puromycin for 10 days.

### 3-D Matrigel Assays

Single cell suspensions of 2×10^4^ MDA-MB-435 cells in 100 µl of complete growth media containing ice cold Matrigel (BD) (1∶1) were plated in triplicates on a 96-well plate. Plates were incubated at 5% CO_2_, 37°C for 30 min to allow the matrigel to solidify, after which 100 µl of complete media was added to each well. The culture media was changed every 3 days. Ten to 14 days after plating, pictures were taken under bright field at 20X magnification using a Leica DMIL inverted microscope.

### Tail Vein Metastasis Assays

Six week-old *Nude (nu/nu)* mice (Charles River Laboratories) were inoculated with 1×10^6^ MDA-MB-435 tumor cells/mouse (in 150 µl of PBS) via tail vein injection. Forty days after tumor cell inoculation, animals were euthanized, and lungs were inflated with India ink, as described [88]. Surface lung nodules and micrometastases were scored in a genotype-blinded fashion using a Leica MZ16 stereomicroscope. Lungs were then paraffin-embedded, and 5 µM sections were stained with hematoxylin-eosin.

### Cell Proliferation Assays

MDA-MBA-435 cells (1.5×10^5^) were seeded in triplicates on 12 well plates on day 0. Cells were grown under subconfluent conditions in complete growth medium containing 1 µg/ml puromycin and split at a ratio of 1∶3 every 3 days. Cell numbers were counted at the time of passaging over a total period of 9 days.

### Chromatin Immunoprecipitation (ChIP)

Cells were grown to 80% confluence and cross-linked with 1% formaldehyde at RT for 10 min. ChIP assays were performed as in [86], except that sonication of cell lysates was performed for 15 pulses of 10 sec with 1 min interval each on ice at 20% power on a Misonix sonicator. Sheared chromatin was immunoprecipitated with 5 µg of antibodies to TBX2 (AB4147; Upstate-Millipore and sc-17880x; Santa Cruz), anti-acetyl Histone 3, or normal rabbit IgG (Upstate-Millipore). PCR primers for amplification of different regions of the mouse *E-cadherin/Cdh1* promoter are listed in Table S1.

### Luciferase Reporter Assays

HC11 were seeded in 12-well tissue culture plates at a density of 2×10^5^ the day prior to transfection. Cells were cotransfected using Lipofectamine 2000 (Invitrogen) with 500 ng of pCDNA3 or pCDNA3-TBX2 expression vectors, 500 ng of pGL2Basic-EcadK1 luciferase reporter construct (containing wild type human *E-Cadherin/CDK1* promoter sequences from −108 to +125/Addgene) [70], and 25 ng of pRL-CMV Renilla plasmid (Promega), which served as normalization control. Forty-eight hours after transfection, cells were harvested and subjected to a Promega Dual Luciferase assay using a Veritas Luminometer.

### Analysis of Published Gene Expression Datasets

Microarray data representing a total of 1107 primary breast tumors from six previously published Affymetrix studies were downloaded from repositories (E-TABM-158, GSE7390, GSE4922, GSE1456, GSE2990, GSE2034) and integrated as described previously using a mean-batch centering method [57]. The NKI [66] and [61] datasets were retrieved from http://microarray-pubs.stanford.edu/would_NKI/explore.html and NCBI GEO (GSE10885). The x-tile method was used to determine the optimal cut-point in Kaplan Meier analysis while correcting for the use of minimum P statistics [89].

### Statistical Analysis

All other data represented in graphs were analyzed by two-sided Student’s *t* or ANOVA tests using GraphPad Prism software. P-values of <0.05 were considered significant.

## Supporting Information

Figure S1 **Ectopic TBX2 induces EMT of MCF10A mammary epithelial cells.** (A) Immunofluorescence analysis of EMT marker expression (40X magnification) shows a reduction and loss of membrane-associated expression of epithelial (green) markers (E-cadherin, ß-catenin, ZO1) with a concomitant gain of mesenchymal (red) marker (N-Cadherin, Vimentin) expression in MCF10A cells stably expressing pCDNA3-TBX2 (+TBX2) as compared to cells expressing pCDNA3 vector (+vector) only. (B) qPCR analysis of TBX2 and EMT marker gene expression using cDNA from the same cells as in (A). Values were normalized to *GAPDH* and fold changes are compared to vector control. The mean ± SEM is shown (n = 3; Student *t*-test). P- values: *p<0.05.
(TIF)

Figure S2 ***TBX2*** **expression in published microarray datasets of primary human breast cancers.** Gene profiling expression data for *TBX2* classified by (A) intrinsic molecular subtypes; (B) Estrogen Receptor alpha (ER) status, as determined by immunohistochemistry; and (C) histological grade, in 1107 tumors from six combined published microarray datasets [57]. (D) *TBX2* expression in the Hennessy et al. dataset comprising 219 tumors including aggressive metaplastic breast tumors [59], [60], [61]. The number of samples in each class and p-values are indicated. NS  =  not significant; v  =  versus.
(TIF)

Figure S3 **Expression and gene amplification of TBX2 in human breast cancer cell lines.** (A–C) Tumor lines are grouped into luminal and basal tumor subtypes according to Neve et al. [90]. (A) Western blot analysis confirms TBX2 protein expression in breast cancer cell lines with known TBX2 gene amplification (open triangle): the luminal Estrogen Receptor (ER)−positive (+) lines MCF7, MDA-MB-361, and BT474; and the basal subtype ER-negative (underlined) breast tumor cell line SUM52. Note that SUM52 is listed as luminal ER+ in the Neve et al. dataset [90] but has been re-classified as basal-subtype triple-negative [62]. Furthermore, TBX2 is overexpressed in the highly invasive basal subgroup metaplastic breast tumor cell lines MDA-MB-157, MDA-MB-435, and weakly in basal subgroup medullary (asterix) HCC1569 tumor cells. (B) qPCR analysis quantifies *TBX2* mRNA expression levels in the tumor cell lines shown in (A). Values were normalized to *GAPDH* mRNA levels and represent fold change as compared to normal human mammary epithelial cells (HMEC). Error bars represent the mean ± SEM (n = 3; Student *t*-test). (C) Comparative genomic hybridization array (aCGH) analysis shows relative gains and losses of the chromosomal region of *TBX2* (17q23) in the selected breast cancer cell lines from two published aCGH studies. Dark grey  =  [90]; light grey  =  [91]. Not all cell lines were represented in both studies, however the relative gains/losses for the TBX2 region between the two studies was significantly correlated (Pearson, R = 0.6, *p = 0.001*) across the overlapping breast tumor cell lines.
(TIF)

Figure S4 **Absence of TBX2 expression in existing** ***BRCA1*** **-deficient breast carcinoma cell lines.** (A) Western blot analysis of endogenous TBX2 protein expression in four *BRCA1^−/−^* breast carcinoma cell lines [92], as indicated. (B) Comparative genomic hybridization array (aCGH) analysis shows no consistent relative gains of the chromosomal region of *TBX2* (17q23) in the selected *BRCA1^−/−^* breast cancer cell lines from two published aCGH studies (Pearson, R = 0.6, *p = 0.001*). Dark grey  =  [90]; light grey  =  [91].
(TIF)

Figure S5 **Reciprocal expression of** ***TBX2*** **and** ***TBX3*** **in normal and neoplastic breast epithelial cell lines.** (A) qPCR analysis of *Tbx2* and *Tbx3* mRNA expression in HC11+vector and HC11+TBX2 cells, and in HC11+TBX2 cells transiently transfected with scrambled siRNAs (siCtrl) or *TBX2* siRNAs (siTBX2) three days post siRNA transfection. Note that, in contrast to *TBX2*, *TBX3* is abundantly expressed in control HC11-vector (+vector) cells. Ectopic expression of TBX2 in HC11 cells (+TBX2) leads to a marked reduction in *Tbx3* mRNA levels, which is reversed by knockdown of exogenous TBX2. (B) qPCR analysis of MDA-MB-435 tumor cells stably expressing non-target control shRNA (shCtrl) or TBX2-specific shRNA (shTBX2) shows low levels of *TBX3* mRNA in endogenously *TBX2*-overexpressing MDA-MB-435 control cells and upregulation of *TBX3* upon TBX2 knockdown. Values were normalized to *GAPDH*. The mean ± SEM is shown (n = 3; Student *t*-test).
(TIF)

Table S1 **List of oligonucleotide sequences separated by assay type.**
(DOC)

## References

[1] Kelemen LE, Wang X, Fredericksen ZS, Pankratz VS, Pharoah PD (2009). Genetic variation in the chromosome 17q23 amplicon and breast cancer risk.. Cancer Epidemiol Biomarkers Prev.

[2] Vance KW, Carreira S, Brosch G, Goding CR (2005). Tbx2 is overexpressed and plays an important role in maintaining proliferation and suppression of senescence in melanomas.. Cancer Res.

[3] Dimova I, Orsetti B, Negre V, Rouge C, Ursule L (2009). Genomic markers for ovarian cancer at chromosomes 1, 8 and 17 revealed by array CGH analysis.. Tumorigenesis.

[4] Liu WK, Jiang XY, Zhang ZX (2010). Expression of PSCA, PIWIL1, and TBX2 in endometrial adenocarcinoma.. Onkologie.

[5] Duo S, Tiao-Dong T, Lei Z, Wei W, Hong-Li S (2009). Expression and clinical significance of tbx2 in pancreatic cancer.. Asian Pac J Cancer Prev.

[6] Barlund M, Monni O, Kononen J, Cornelison R, Torhorst J (2000). Multiple genes at 17q23 undergo amplification and overexpression in breast cancer.. Cancer Res.

[7] Sinclair CS, Adem C, Naderi A, Soderberg CL, Johnson M (2002). TBX2 is preferentially amplified in BRCA1- and BRCA2-related breast tumors.. Cancer Res.

[8] Andersen CL, Monni O, Wagner U, Kononen J, Barlund M (2002). High-Throughput Copy Number analysis of 17q23 in 3520 Tissue Specimens by Fluorescence in Situ Hybridization to Tissue Microarrays.. Am J Pathol.

[9] Jacobs JJ, Keblusek P, Robanus-Maandag E, Kristel P, Lingbeek M (2000). Senescence bypass screen identifies TBX2, which represses Cdkn2a (p19(ARF)) and is amplified in a subset of human breast cancers.. Nat Genet.

[10] Redmond KL, Crawford NT, Farmer H, D’Costa ZC, O’Brien GJ (2010). T-box 2 represses NDRG1 through an EGR1-dependent mechanism to drive the proliferation of breast cancer cells.. Oncogene.

[11] Bollag RJ, Siegfried Z, Cebra-Thomas JA, Garvey N, Davison EM (1994). An Ancient family of embryonically expressed mouse genes sharing a conserved protein motif with the T locus.. Nat Genet.

[12] Abrahams A, Parker MI, Prince S (2010). The T-box transcription factor Tbx2: its role in development and possible implication in cancer.. IUBMB Life.

[13] Naiche LA, Harrelson Z, Kelly RG, Papaioannou VE (2005). T-box genes in vertebrate development.. Annu Rev Genet.

[14] Carreira S, Dexter TJ, Yavuzer U, Easty DJ, Goding CR (1998). Brachyury-related transcription factor Tbx2 and repression of the melanocyte-specific TRP-1 promoter.. Mol Cell Biol.

[15] Lingbeek ME, Jacobs JJ, van Lohuizen M (2002). The T-box repressors TBX2 and TBX3 specifically regulate the tumor suppressor gene p14ARF via a variant T-site in the initiator.. J Biol Chem.

[16] Paxton C, Zhao H, Chin Y, Langner K, Reecy J (2002). Murine Tbx2 contains domains that activate and repress gene transcription.. Gene.

[17] Suzuki T, Takeuchi J, Koshiba-Takeuchi K, Ogura T (2004). Tbx Genes Specify Posterior Digit Identity through Shh and BMP Signaling.. Dev Cell.

[18] Harrelson Z, Kelly RG, Goldin SN, Gibson-Brown JJ, Bollag RJ (2004). Tbx2 is essential for patterning the atrioventricular canal and for morphogenesis of the outflow tract during heart development.. Development.

[19] Cho GS, Choi SC, Park EC, Han JK (2011). Role of Tbx2 in defining the territory of the pronephric nephron.. Development.

[20] Fong SH, Emelyanov A, Teh C, Korzh V (2005). Wnt signalling mediated by Tbx2b regulates cell migration during formation of the neural plate.. Development.

[21] Manning L, Ohyama K, Saeger B, Hatano O, Wilson SA (2006). Regional morphogenesis in the hypothalamus: a BMP-Tbx2 pathway coordinates fate and proliferation through Shh downregulation.. Dev Cell.

[22] Behesti H, Papaioannou VE, Sowden JC (2009). Loss of Tbx2 delays optic vesicle invagination leading to small optic cups.. Dev Biol.

[23] Chapman DL, Garvey N, Hancock S, Alexiou M, Agulnik SI (1996). Expression of the T-box family genes, Tbx1-Tbx5, during early mouse development.. Dev Dyn.

[24] Jerome-Majewska LA, Jenkins GP, Ernstoff E, Zindy F, Sherr CJ (2005). Tbx3, the ulnar-mammary syndrome gene, and Tbx2 interact in mammary gland development through a p19Arf/p53-independent pathway.. Dev Dyn.

[25] Vance KW, Shaw HM, Rodriguez M, Ott S, Goding CR (2010). The retinoblastoma protein modulates tbx2 functional specificity.. Mol Biol Cell.

[26] Martin N, Benhamed M, Nacerddine K, Demarque MD, van Lohuizen M (2011). Physical and functional interaction between PML and TBX2 in the establishment of cellular senescence.. Embo J.

[27] Ribeiro I, Kawakami Y, Buscher D, Raya A, Rodriguez-Leon J (2007). Tbx2 and Tbx3 regulate the dynamics of cell proliferation during heart remodeling.. PLoS One.

[28] Davis E, Teng H, Bilican B, Parker MI, Liu B (2008). Ectopic Tbx2 expression results in polyploidy and cisplatin resistance.. Oncogene.

[29] Serrano M, Lin AW, McCurrach ME, Beach D, Lowe SW (1997). Oncogenic ras provokes premature cell senescence associated with accumulation of p53 and p16INK4a.. Cell.

[30] Prince S, Carreira S, Vance KW, Abrahams A, Goding CR (2004). Tbx2 directly represses the expression of the p21(WAF1) cyclin-dependent kinase inhibitor.. Cancer Res.

[31] Ismail A, Bateman A (2009). Expression of TBX2 promotes anchorage-independent growth and survival in the p53-negative SW13 adrenocortical carcinoma.. Cancer Lett.

[32] Vormer TL, Foijer F, Wielders CL, te Riele H (2008). Anchorage-independent growth of pocket protein-deficient murine fibroblasts requires bypass of G2 arrest and can be accomplished by expression of TBX2.. Mol Cell Biol.

[33] Thiery JP, Acloque H, Huang RY, Nieto MA (2009). Epithelial-mesenchymal transitions in development and disease.. Cell.

[34] Hay ED (2005). The mesenchymal cell, its role in the embryo, and the remarkable signaling mechanisms that create it.. Dev Dyn.

[35] Oft M, Peli J, Rudaz C, Schwarz H, Beug H (1996). TGF-beta1 and Ha-Ras collaborate in modulating the phenotypic plasticity and invasiveness of epithelial tumor cells.. Genes Dev.

[36] Batlle E, Sancho E, Franci C, Dominguez D, Monfar M (2000). The transcription factor snail is a repressor of E-cadherin gene expression in epithelial tumour cells.. Nat Cell Biol.

[37] Cano A, Perez-Moreno MA, Rodrigo I, Locascio A, Blanco MJ (2000). The transcription factor snail controls epithelial-mesenchymal transitions by repressing E-cadherin expression.. Nat Cell Biol.

[38] Eger A, Aigner K, Sonderegger S, Dampier B, Oehler S (2005). DeltaEF1 is a transcriptional repressor of E-cadherin and regulates epithelial plasticity in breast cancer cells.. Oncogene.

[39] Comijn J, Berx G, Vermassen P, Verschueren K, van Grunsven L (2001). The two-handed E box binding zinc finger protein SIP1 downregulates E-cadherin and induces invasion.. Mol Cell.

[40] Yang J, Mani SA, Donaher JL, Ramaswamy S, Itzykson RA (2004). Twist, a master regulator of morphogenesis, plays an essential role in tumor metastasis.. Cell.

[41] Ansieau S, Bastid J, Doreau A, Morel AP, Bouchet BP (2008). Induction of EMT by twist proteins as a collateral effect of tumor-promoting inactivation of premature senescence.. Cancer Cell.

[42] Liu Y, El-Naggar S, Darling DS, Higashi Y, Dean DC (2008). Zeb1 links epithelial-mesenchymal transition and cellular senescence.. Development.

[43] Hartwell KA, Muir B, Reinhardt F, Carpenter AE, Sgroi DC (2006). The Spemann organizer gene, Goosecoid, promotes tumor metastasis.. Proc Natl Acad Sci U S A.

[44] Yu M, Smolen GA, Zhang J, Wittner B, Schott BJ (2009). A developmentally regulated inducer of EMT, LBX1, contributes to breast cancer progression.. Genes Dev.

[45] Micalizzi DS, Christensen KL, Jedlicka P, Coletta RD, Baron AE (2009). The Six1 homeoprotein induces human mammary carcinoma cells to undergo epithelial-mesenchymal transition and metastasis in mice through increasing TGF-beta signaling.. J Clin Invest.

[46] Mani SA, Yang J, Brooks M, Schwaninger G, Zhou A (2007). Mesenchyme Forkhead 1 (FOXC2) plays a key role in metastasis and is associated with aggressive basal-like breast cancers.. Proc Natl Acad Sci U S A.

[47] Briegel KJ (2006). Embryonic transcription factors in human breast cancer.. IUBMB Life.

[48] Barrallo-Gimeno A, Nieto MA (2005). The Snail genes as inducers of cell movement and survival: implications in development and cancer.. Development.

[49] Yang J, Weinberg RA (2008). Epithelial-mesenchymal transition: at the crossroads of development and tumor metastasis.. Dev Cell.

[50] Vandewalle C, Van Roy F, Berx G (2009). The role of the ZEB family of transcription factors in development and disease.. Cell Mol Life Sci.

[51] Mani SA, Guo W, Liao MJ, Eaton EN, Ayyanan A (2008). The epithelial-mesenchymal transition generates cells with properties of stem cells.. Cell.

[52] Morel AP, Lievre M, Thomas C, Hinkal G, Ansieau S (2008). Generation of breast cancer stem cells through epithelial-mesenchymal transition.. PLoS One.

[53] Creighton CJ, Li X, Landis M, Dixon JM, Neumeister VM (2009). Residual breast cancers after conventional therapy display mesenchymal as well as tumor-initiating features.. Proc Natl Acad Sci U S A.

[54] Moody SE, Perez D, Pan TC, Sarkisian CJ, Portocarrero CP (2005). The transcriptional repressor Snail promotes mammary tumor recurrence.. Cancer Cell.

[55] Taneja P, Maglic D, Kai F, Zhu S, Kendig RD (2010). Classical and Novel Prognostic Markers for Breast Cancer and their Clinical Significance.. Clin Med Insights Oncol.

[56] Lindley LE, Briegel KJ (2010). Molecular characterization of TGFbeta-induced epithelial-mesenchymal transition in normal finite lifespan human mammary epithelial cells.. Biochem Biophys Res Commun.

[57] Sims AH, Smethurst GJ, Hey Y, Okoniewski MJ, Pepper SD (2008). The removal of multiplicative, systematic bias allows integration of breast cancer gene expression datasets - improving meta-analysis and prediction of prognosis.. BMC Medical Genomics.

[58] Sorlie T, Tibshirani R, Parker J, Hastie T, Marron JS (2003). Repeated observation of breast tumor subtypes in independent gene expression data sets.. Proc Natl Acad Sci U S A.

[59] Prat A, Parker JS, Karginova O, Fan C, Livasy C (2010). Phenotypic and molecular characterization of the claudin-low intrinsic subtype of breast cancer.. Breast Cancer Res.

[60] Taube JH, Herschkowitz JI, Komurov K, Zhou AY, Gupta S (2010). Core epithelial-to-mesenchymal transition interactome gene-expression signature is associated with claudin-low and metaplastic breast cancer subtypes.. Proc Natl Acad Sci U S A.

[61] Hennessy BT, Gonzalez-Angulo AM, Stemke-Hale K, Gilcrease MZ, Krishnamurthy S (2009). Characterization of a naturally occurring breast cancer subset enriched in epithelial-to-mesenchymal transition and stem cell characteristics.. Cancer Res.

[62] Turner N, Lambros MB, Horlings HM, Pearson A, Sharpe R (2010). Integrative molecular profiling of triple negative breast cancers identifies amplicon drivers and potential therapeutic targets.. Oncogene.

[63] Cailleau R, Olive M, Cruciger QV (1978). Long-term human breast carcinoma cell lines of metastatic origin: preliminary characterization.. In Vitro.

[64] Hollestelle A, Nagel JH, Smid M, Lam S, Elstrodt F (2010). Distinct gene mutation profiles among luminal-type and basal-type breast cancer cell lines.. Breast Cancer Res Treat.

[65] Price JE (1996). Metastasis from human breast cancer cell lines.. Breast Cancer Res Treat.

[66] van de Vijver MJ, He YD, van’t Veer LJ, Dai H, Hart AA (2002). A gene-expression signature as a predictor of survival in breast cancer.. N Engl J Med.

[67] Christofori G (2003). Changing neighbours, changing behaviour: cell adhesion molecule-mediated signalling during tumour progression.. Embo J.

[68] Onder TT, Gupta PB, Mani SA, Yang J, Lander ES (2008). Loss of E-cadherin promotes metastasis via multiple downstream transcriptional pathways.. Cancer Res.

[69] Rodriguez M, Aladowicz E, Lanfrancone L, Goding CR (2008). Tbx3 represses E-cadherin expression and enhances melanoma invasiveness.. Cancer Res.

[70] Hajra KM, Ji X, Fearon ER (1999). Extinction of E-cadherin expression in breast cancer via a dominant repression pathway acting on proximal promoter elements.. Oncogene.

[71] Christoffels VM, Hoogaars WM, Tessari A, Clout DE, Moorman AF (2004). T-box transcription factor Tbx2 represses differentiation and formation of the cardiac chambers.. Dev Dyn.

[72] Shirai M, Imanaka-Yoshida K, Schneider MD, Schwartz RJ, Morisaki T (2009). T-box 2, a mediator of Bmp-Smad signaling, induced hyaluronan synthase 2 and Tgfbeta2 expression and endocardial cushion formation.. Proc Natl Acad Sci U S A.

[73] Trujillo KA, Heaphy CM, Mai M, Vargas KM, Jones AC (2010). Markers of fibrosis and epithelial to mesenchymal transition demonstrate field cancerization in histologically normal tissue adjacent to breast tumors.. Int J Cancer.

[74] Peres J, Davis E, Mowla S, Bennett DC, Li JA (2011). The Highly Homologous T-Box Transcription Factors, TBX2 and TBX3, Have Distinct Roles in the Oncogenic Process.. Genes Cancer.

[75] Brummelkamp TR, Kortlever RM, Lingbeek M, Trettel F, MacDonald ME (2002). TBX-3, the gene mutated in Ulnar-Mammary Syndrome, is a negative regulator of p19ARF and inhibits senescence.. J Biol Chem.

[76] Kumar S, Park SH, Cieply B, Schupp J, Killiam E (2011). A pathway for the control of anoikis sensitivity by E-cadherin and epithelial-to-mesenchymal transition.. Mol Cell Biol.

[77] Fan W, Huang X, Chen C, Gray J, Huang T (2004). TBX3 and its isoform TBX3+2a are functionally distinctive in inhibition of senescence and are overexpressed in a subset of breast cancer cell lines.. Cancer Res.

[78] Fillmore CM, Gupta PB, Rudnick JA, Caballero S, Keller PJ (2010). Estrogen expands breast cancer stem-like cells through paracrine FGF/Tbx3 signaling.. Proc Natl Acad Sci U S A.

[79] Lim E, Wu D, Pal B, Bouras T, Asselin-Labat ML (2010). Transcriptome analyses of mouse and human mammary cell subpopulations reveal multiple conserved genes and pathways.. Breast Cancer Res.

[80] Bamshad M, Lin RC, Law DJ, Watkins WC, Krakowiak PA (1997). Mutations in human TBX3 alter limb, apocrine and genital development in ulnar-mammary syndrome.. Nat Genet.

[81] Davenport TG, Jerome-Majewska LA, Papaioannou VE (2003). Mammary gland, limb and yolk sac defects in mice lacking Tbx3, the gene mutated in human ulnar mammary syndrome.. Development.

[82] Chia SK, Speers CH, Bryce CJ, Hayes MM, Olivotto IA (2004). Ten-year outcomes in a population-based cohort of node-negative, lymphatic, and vascular invasion-negative early breast cancers without adjuvant systemic therapies.. J Clin Oncol.

[83] Fisher B, Jeong JH, Bryant J, Anderson S, Dignam J (2004). Treatment of lymph-node-negative, oestrogen-receptor-positive breast cancer: long-term findings from National Surgical Adjuvant Breast and Bowel Project randomised clinical trials.. Lancet.

[84] Ball RK, Friis RR, Schoenenberger CA, Doppler W, Groner B (1988). Prolactin regulation of b-casein gene expression and of a cytosolic 120-kd protein in a cloned mouse mammary epithelial cell line.. EMBO J.

[85] Chen J, Zhong Q, Wang J, Cameron RS, Borke JL (2001). Microarray analysis of Tbx2-directed gene expression: a possible role in osteogenesis.. Mol Cell Endocrinol.

[86] Rieger ME, Sims AH, Coats ER, Clarke RB, Briegel KJ (2010). The embryonic transcription cofactor LBH is a direct target of the Wnt signaling pathway in epithelial development and in aggressive basal subtype breast cancers.. Mol Cell Biol.

[87] Liang CC, Park AY, Guan JL (2007). In vitro scratch assay: a convenient and inexpensive method for analysis of cell migration in vitro.. Nat Protoc.

[88] Williams TM, Medina F, Badano I, Hazan RB, Hutchinson J (2004). Caveolin-1 gene disruption promotes mammary tumorigenesis and dramatically enhances lung metastasis in vivo. Role of Cav-1 in cell invasiveness and matrix metalloproteinase (MMP-2/9) secretion.. J Biol Chem.

[89] Camp RL, Dolled-Filhart M, Rimm DL (2004). X-tile: a new bio-informatics tool for biomarker assessment and outcome-based cut-point optimization.. Clin Cancer Res.

[90] Neve RM, Chin K, Fridlyand J, Yeh J, Baehner FL (2006). A collection of breast cancer cell lines for the study of functionally distinct cancer subtypes.. Cancer Cell.

[91] Chin SF, Teschendorff AE, Marioni JC, Wang Y, Barbosa-Morais NL (2007). High-resolution aCGH and expression profiling identifies a novel genomic subtype of ER negative breast cancer.. Genome Biol.

[92] Elstrodt F, Hollestelle A, Nagel JH, Gorin M, Wasielewski M (2006). BRCA1 mutation analysis of 41 human breast cancer cell lines reveals three new deleterious mutants.. Cancer Res.

