# Supplementary figures and images for: The T Box Transcription Factor TBX2 Promotes Epithelial-Mesenchymal Transition and Invasion of Normal and Malignant Breast Epithelial Cells
Source: PLoS One. 2012 Jul 23;7(7):e41355. doi: 10.1371/journal.pone.0041355 (PMC3402503; doi:10.1371/journal.pone.0041355)

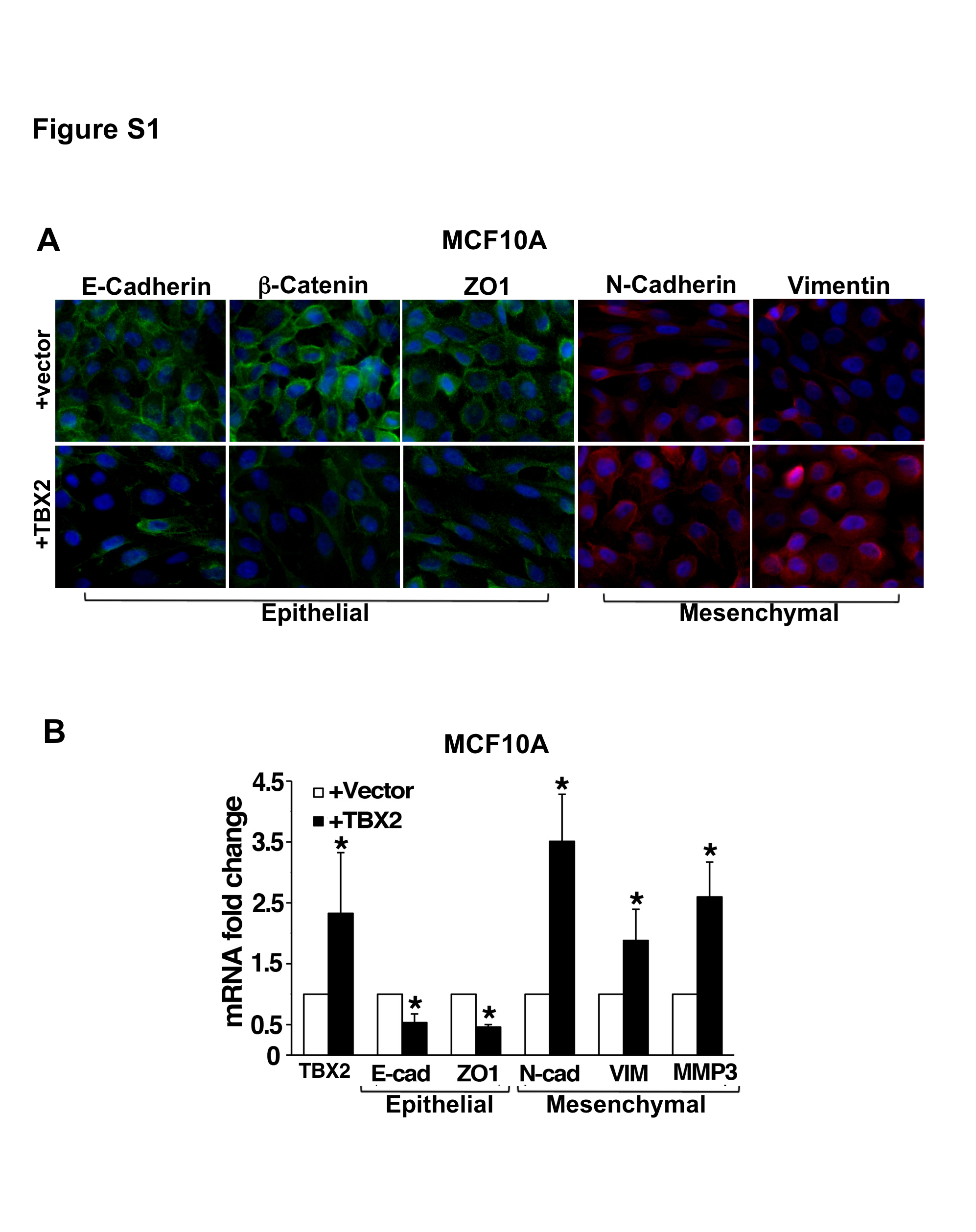

Supplement: Figure S1 — Ectopic TBX2 induces EMT of MCF10A mammary epithelial cells. (A) Immunofluorescence analysis of EMT marker expression (40X magnification) shows a reduction and loss of membrane-associated expression of epithelial (green) markers (E-cadherin, ß-catenin, ZO1) with a concomitant gain of mesenchymal (red) marker (N-Cadherin, Vimentin) expression in MCF10A cells stably expressing pCDNA3-TBX2 (+TBX2) as compared to cells expressing pCDNA3 vector (+vector) only. (B) qPCR analysis of TBX2 and EMT marker gene expression using cDNA from the same cells as in (A). Values were normalized to GAPDH and fold changes are compared to vector control. The mean ± SEM is shown (n = 3; Student t-test). P- values: *p<0.05. (TIF) [file pone.0041355.s001.tif]

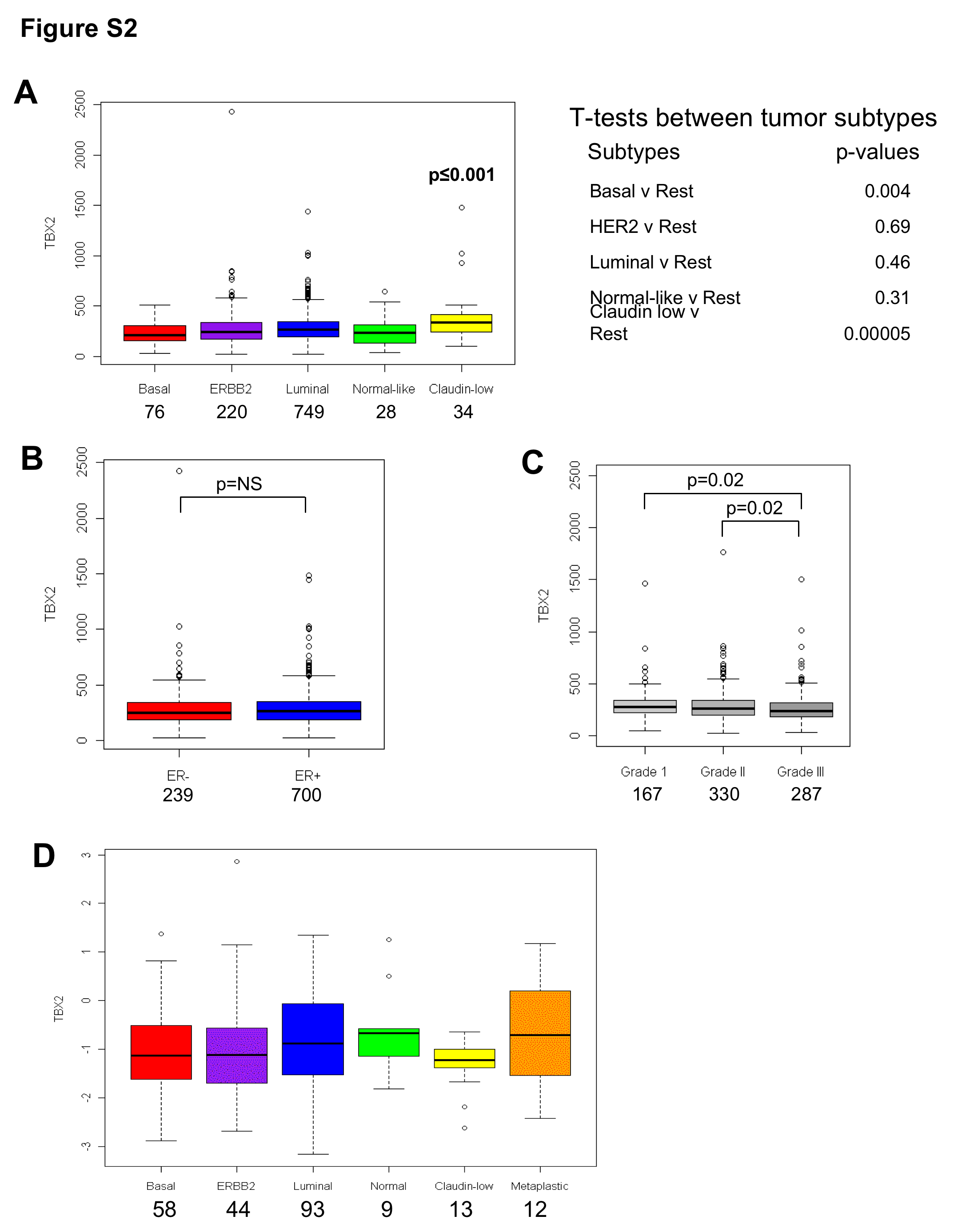

Supplement: Figure S2 — TBX2 expression in published microarray datasets of primary human breast cancers. Gene profiling expression data for TBX2 classified by (A) intrinsic molecular subtypes; (B) Estrogen Receptor alpha (ER) status, as determined by immunohistochemistry; and (C) histological grade, in 1107 tumors from six combined published microarray datasets [57]. (D) TBX2 expression in the Hennessy et al. dataset comprising 219 tumors including aggressive metaplastic breast tumors [59], [60], [61]. The number of samples in each class and p-values are indicated. NS = not significant; v = versus. (TIF) [file pone.0041355.s002.tif]

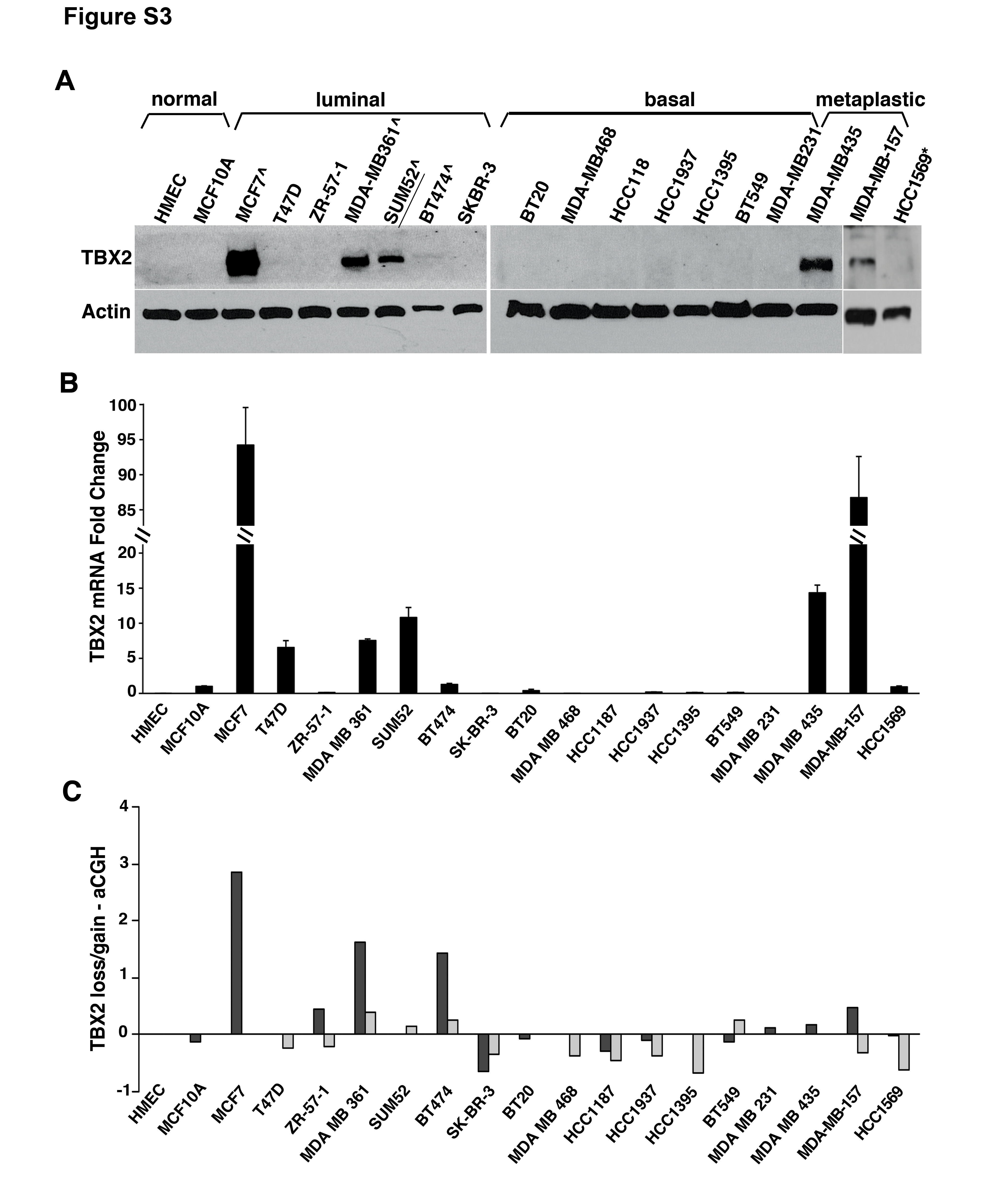

Supplement: Figure S3 — Expression and gene amplification of TBX2 in human breast cancer cell lines. (A–C) Tumor lines are grouped into luminal and basal tumor subtypes according to Neve et al. [90]. (A) Western blot analysis confirms TBX2 protein expression in breast cancer cell lines with known TBX2 gene amplification (open triangle): the luminal Estrogen Receptor (ER)−positive (+) lines MCF7, MDA-MB-361, and BT474; and the basal subtype ER-negative (underlined) breast tumor cell line SUM52. Note that SUM52 is listed as luminal ER+ in the Neve et al. dataset [90] but has been re-classified as basal-subtype triple-negative [62]. Furthermore, TBX2 is overexpressed in the highly invasive basal subgroup metaplastic breast tumor cell lines MDA-MB-157, MDA-MB-435, and weakly in basal subgroup medullary (asterix) HCC1569 tumor cells. (B) qPCR analysis quantifies TBX2 mRNA expression levels in the tumor cell lines shown in (A). Values were normalized to GAPDH mRNA levels and represent fold change as compared to normal human mammary epithelial cells (HMEC). Error bars represent the mean ± SEM (n = 3; Student t-test). (C) Comparative genomic hybridization array (aCGH) analysis shows relative gains and losses of the chromosomal region of TBX2 (17q23) in the selected breast cancer cell lines from two published aCGH studies. Dark grey = [90]; light grey = [91]. Not all cell lines were represented in both studies, however the relative gains/losses for the TBX2 region between the two studies was significantly correlated (Pearson, R = 0.6, p = 0.001) across the overlapping breast tumor cell lines. (TIF) [file pone.0041355.s003.tif]

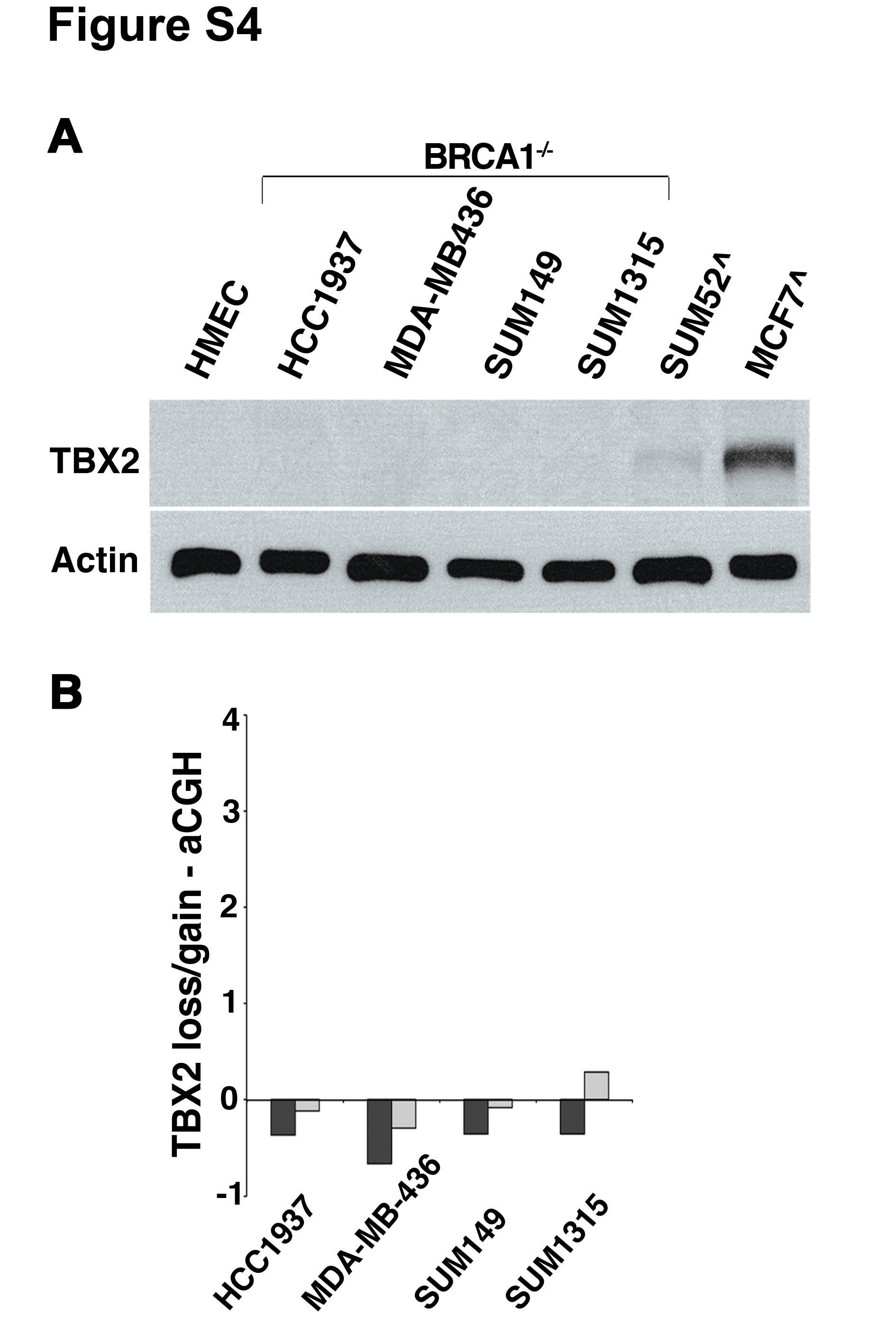

Supplement: Figure S4 — Absence of TBX2 expression in existing BRCA1 -deficient breast carcinoma cell lines. (A) Western blot analysis of endogenous TBX2 protein expression in four BRCA1−/− breast carcinoma cell lines [92], as indicated. (B) Comparative genomic hybridization array (aCGH) analysis shows no consistent relative gains of the chromosomal region of TBX2 (17q23) in the selected BRCA1−/− breast cancer cell lines from two published aCGH studies (Pearson, R = 0.6, p = 0.001). Dark grey = [90]; light grey = [91]. (TIF) [file pone.0041355.s004.tif]

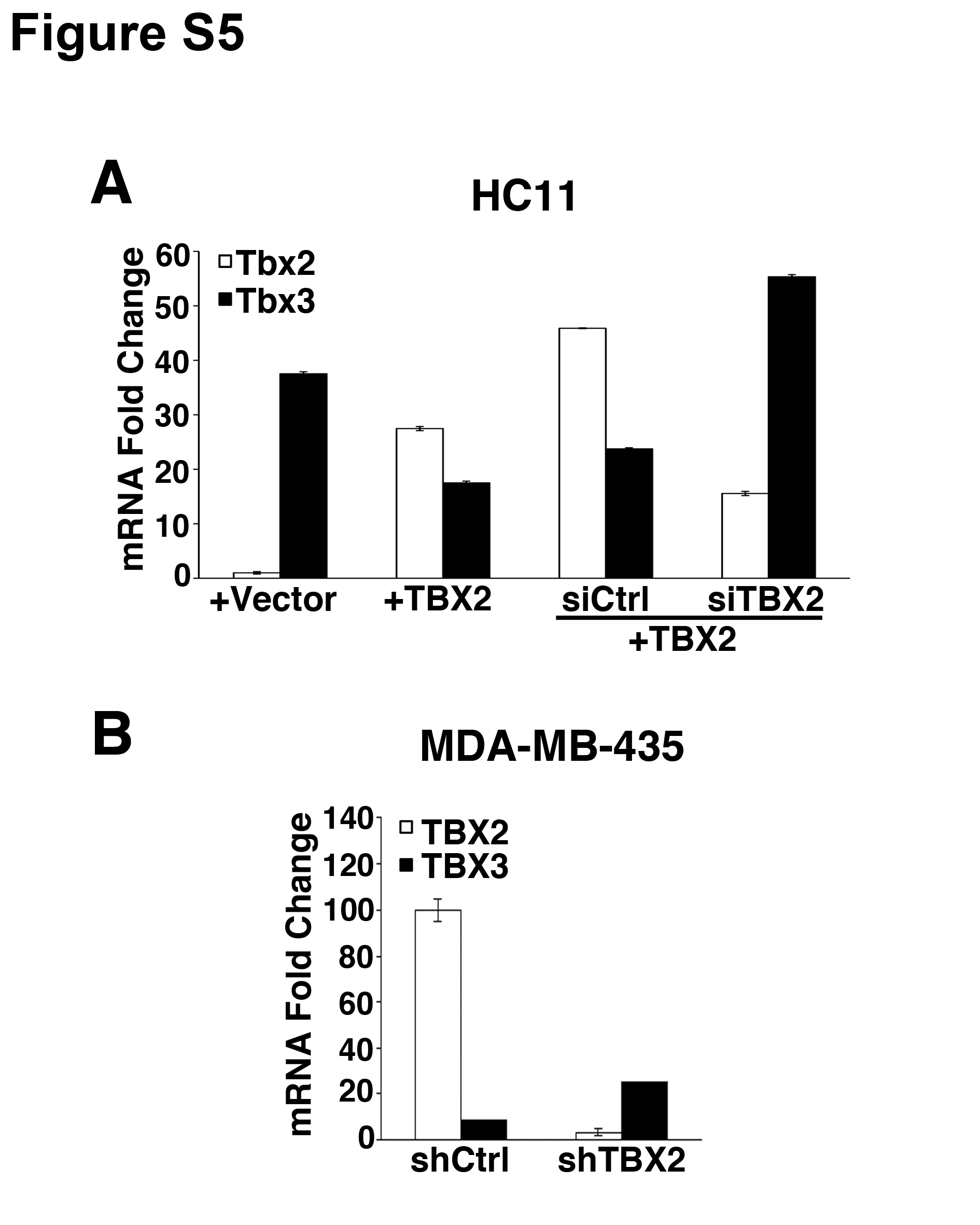

Supplement: Figure S5 — Reciprocal expression of TBX2 and TBX3 in normal and neoplastic breast epithelial cell lines. (A) qPCR analysis of Tbx2 and Tbx3 mRNA expression in HC11+vector and HC11+TBX2 cells, and in HC11+TBX2 cells transiently transfected with scrambled siRNAs (siCtrl) or TBX2 siRNAs (siTBX2) three days post siRNA transfection. Note that, in contrast to TBX2, TBX3 is abundantly expressed in control HC11-vector (+vector) cells. Ectopic expression of TBX2 in HC11 cells (+TBX2) leads to a marked reduction in Tbx3 mRNA levels, which is reversed by knockdown of exogenous TBX2. (B) qPCR analysis of MDA-MB-435 tumor cells stably expressing non-target control shRNA (shCtrl) or TBX2-specific shRNA (shTBX2) shows low levels of TBX3 mRNA in endogenously TBX2-overexpressing MDA-MB-435 control cells and upregulation of TBX3 upon TBX2 knockdown. Values were normalized to GAPDH. The mean ± SEM is shown (n = 3; Student t-test). (TIF) [file pone.0041355.s005.tif]
