# Supplementary material for: The T Box Transcription Factor TBX2 Promotes Epithelial-Mesenchymal Transition and Invasion of Normal and Malignant Breast Epithelial Cells
Source: PLoS One. 2012 Jul 23;7(7):e41355. doi: 10.1371/journal.pone.0041355 (PMC3402503; doi:10.1371/journal.pone.0041355)
Supplement: Table S1 — List of oligonucleotide sequences separated by assay type. (DOC) [file pone.0041355.s006.doc]

**Table S1: List of oligonucleotide sequences separated by assay type**

| **qPCR primers** | | | |
| --- | --- | --- | --- |
| **Gene** | **Species** | **Primer** | **Sequence (5’-3’)** |
| *Tbx2* | Human/Mouse | Forward | CCCTTCCCGTTCCACCTC |
|  |  | Reverse | ACTAGTGGCGGGCAAAGC |
| *Dsp* | Mouse | Forward | AGTTCACCAAACGCCTCACGGG |
|  |  | Reverse | TTGTCCAGCTGCCAGCGGTAGT |
| *E-cadherin* | Mouse | Forward | CTCAGTGTTTGCTCGGCGTC |
|  |  | Reverse | AATCTCACTCTGCCCAGGACATG |
| *E-Cadherin* | Human | Forward | AATTCCTGCCATTCTGGGGA |
|  |  | Reverse | tcttctccgcctccttcttc |
| *Fibronectin* | Human | Forward | CAGTGGGAGACCTCGAGAAG |
|  |  | Reverse | TCCCTCGGAACATCAGAAAC |
| *Mmp3* | Mouse | Forward | TGCTGTCTTTGAAGCATTTGGGTT |
|  |  | Reverse | GCACTTCCTTTCACAAAGACTCAGA |
| *MMP3* | Human | Forward | TCAGTCCCTCTATGGACCTCCCCC |
|  |  | Reverse | AGGGATTTGCGCCAAAAGTGCCT |
| *N-cadherin* | Mouse | Forward | CTGCTCAGGACCCCGATCGA |
|  |  | Reverse | GGCGGGATTCCATTGTCAGAAG |
| *N-cadherin* | Human | Forward | TGAGCCTGAAGCCAACCTTA |
|  |  | Reverse | AGGTCCCCTGGAGTTTTCTG |
| *Vimentin* | Mouse | Forward | GGCTGCGAGAGAAATTGCAGGAGG |
|  |  | Reverse | GCTGTTCCTGAATCTGGGCCTGC |
| *Vimentin* | Human | Forward | AGCTAACCAACGACAAAGCC |
|  |  | Reverse | TCCACTTTGCGTTCAAGGTC |
| *ZO-1* | Mouse | Forward | CCCCCGGAGTCTGCCATTAC |
|  |  | Reverse | TGGAGATGAGGCTTCTGCTTTCTGT |
| *ZO-1* | Human | Forward | CGAGTTGCAATGGTTAACGGA |
|  |  | Reverse | TCAGGATCAGGACGACTTACTGG |
| *Tbx3* | Mouse | Forward | CAACTCTCGGTGGATGGTGGC |
|  |  | Reverse | TCGCTTGGGAAGGCCAAAGTAAAT |
| *TBX3* | Human | Forward | TCCATGAGGGTGTTTGATGA |
|  |  | Reverse | CCATGCTCCTCTTTGCTCTC |
| *Gapdh* | Mouse | Forward | GACCCCTTCATTGACCTCAAC |
|  |  | Reverse | CTTCTCCATGGTGGTGAAGA |
| *GAPDH* | Human | Forward | ATCAAGTGGGGCGATGCTG |
|  |  | Reverse | ACCCATGACGAACATGGGG |
| **ChIP primer sequences for mouse *E-cadherin/Cdh1*** | | | |
| **Name** | | **Primer** | **Sequence (5’-3’)** |
| Proximal (-131/+61) | | Forward | AGACAGGGGTGGAGGAAGTT |
|  | | Reverse | GGGCAGGAGTCTAGCAGAAG |
| Half T site (-726/-573) | | Forward | CATGCTGGGCTACATAGCAA |
|  | | Reverse | TGGGCCTGGAATTGTCTTAG |
| Distal (-1299/-1119) | | Forward | AGCGGTTGAGAGCATTGACT |
|  | | Reverse | CAGCCCTCCTCATCAGTTGT |
